# CD8^+^ lymphocyte control of SIV infection during antiretroviral therapy

**DOI:** 10.1371/journal.ppat.1007350

**Published:** 2018-10-11

**Authors:** Youfang Cao, Emily K. Cartwright, Guido Silvestri, Alan S. Perelson

**Affiliations:** 1 Theoretical Biology and Biophysics (T-6), Los Alamos National Laboratory, Los Alamos, NM, United States of America; 2 Center for Nonlinear Studies (CNLS), Los Alamos National Laboratory, Los Alamos, NM, United States of America; 3 Emory Vaccine Center and Yerkes National Primate Research Center, Emory University, Atlanta, GA, United States of America; Vaccine Research Center, UNITED STATES

## Abstract

CD8^+^ lymphocytes play an important role in suppressing *in vivo* viral replication in HIV infection. However, both the extent to which and the mechanisms by which CD8^+^ lymphocytes contribute to viral control are not completely understood. A recent experiment depleted CD8^+^ lymphocytes in simian immunodeficiency virus (SIV)-infected rhesus macaques (RMs) on antiretroviral treatment (ART) to study the role of CD8^+^ lymphocytes. CD8^+^ lymphocytes depletion resulted in temporary plasma viremia in all studied RMs. Viral control was restored when CD8^+^ lymphocytes repopulated. We developed a viral dynamic model to fit the viral load (VL) data from the CD8 depletion experiment. We explicitly modeled the dynamics of the latent reservoir and the SIV-specific effector cell population including their exhaustion and their potential cytolytic and noncytolytic functions. We found that the latent reservoir significantly contributes to the size of the peak VL after CD8 depletion, while drug efficacy plays a lesser role. Our model suggests that the overall CD8^+^ lymphocyte cytolytic killing rate is dynamically changing depending on the levels of antigen-induced effector cell activation and exhaustion. Based on estimated parameters, our model suggests that before ART or without ART the overall CD8 cytolytic killing rate is small due to exhaustion. However, after the start of ART, the overall CD8 cytolytic killing rate increases due to an expansion of SIV-specific CD8 effector cells. Further, we estimate that the cytolytic killing rate can be significantly larger than the cytopathic death rate in some animals during the second phase of ART-induced viral decay. Lastly, our model provides a new explanation for the puzzling findings by Klatt et al. and Wong et al. that CD8 depletion done immediately before ART has no noticeable effect on the first phase viral decay slope seen after ART initiation Overall, by incorporating effector cells and their exhaustion, our model can explain the effects of CD8 depletion on VL during ART, reveals a detailed dynamic role of CD8^+^ lymphocytes in controlling viral infection, and provides a unified explanation for CD8 depletion experimental data.

## Author summary

CD8^+^ lymphocytes play an important role in suppressing *in vivo* viral replication in HIV infection. However, both the extent to which and the mechanisms by which CD8^+^ lymphocytes contribute to viral control are not completely understood. By mathematically modeling data from a recent CD8 depletion experiment done in antiretroviral (ART) treated animals, our results suggest that the overall CD8^+^ lymphocyte cytolytic killing rate is dynamically changing depending on the levels of antigen-induced effector cell activation and exhaustion, i.e. before ART or without ART the overall CD8 cytolytic killing rate is small due to exhaustion. However, after the start of ART, the overall CD8 cytolytic killing rate increases due to an expansion of SIV-specific CD8 effector cells. By incorporating effector cells and their exhaustion, our model explains the effects on viral load of CD8 depletion done before ART or during ART, reveals a detailed dynamic role of CD8^+^ lymphocytes in controlling viral infection, and provides a unified explanation for CD8 depletion experimental data.

## Introduction

Current antiretroviral treatment (ART) can suppress viral replication in HIV infected patients, but it cannot eliminate the infection due to the persistent latent reservoir. Once ART is interrupted, the majority of patients exhibit viral rebound and lose viral control. To eliminate HIV infection or control HIV replication to low levels leading to functional cure, HIV infected cells need to be eliminated. These infected cells can be killed by viral cytopathic effects or through cell-mediated killing by cytotoxic CD8^+^ T cells, CD4^+^ T cells, or NK cells. CD8^+^ lymphocytes can also reduce viral production through non-cytolytic effects.

CD8^+^ lymphocytes play an important role in suppressing *in vivo* viral replication in HIV infection. However, the detailed mechanisms of action and relative contributions of CD8^+^ lymphocytes in controlling viral infection are not fully understood[1]. CD8 depletion experiments have shown that in untreated chronically infected rhesus macaques (RMs), viral load (VL) increases after CD8 depletion, and the VL returns to baseline after CD8^+^ lymphocytes are repopulated[2–4], suggesting a strong role of CD8^+^ lymphocytes in controlling chronic SIV infection. Klatt et al. [5] and Wong et al. [6] showed that the first phase slope of VL decay after ART was initiated was not changed in CD8 depleted animals suggesting that CD8^+^ lymphocytes may not be killing infected cells. This explanation has been controversial and other explanations have been proposed [5–9]. Klatt et al. [5] and Wong et al. [6] suggested that CD8^+^ lymphocytes might be killing infected cells during the eclipse phase. Because cells in the eclipse phase are not yet producing virus such killing would not influence the first phase viral decay rate. Gadhamsetty et al. [8] developed a model including an eclipse phase that suggested that under certain circumstances the slope of VL decline after ART initiation is not determined by the death rate of productively infected cells. In this paper we will provide yet another explanation.

In a recent CD8 depletion study by Cartwright et al.[10], CD8^+^ lymphocytes in ART-treated SIV-infected rhesus macaques were depleted after the VL was driven to near or below the detection limit by ART to study the role of CD8^+^ lymphocytes in viral control. CD8 depletion during ART resulted in a temporary increase of plasma viremia in all studied RMs, and viral control was regained when the CD8^+^ lymphocytes population recovered. In this study, we model the data from this CD8 depletion experiment using different mathematical models to explore the mechanisms of action of CD8^+^ lymphocytes in viral control. We characterize the relative roles of viral cytopathic cell death and CD8 cell cytolytic killing as well as non-cytolytic effects in controlling viral replication. Interestingly, our model suggests a dynamic role of CD8 lymphocyte effects, which are small before or without ART due to CD8 lymphocyte exhaustion, but which can significantly increase after ART initiation and VL decease. Specifically, our model analysis of the data presented in Cartwright et al. [10] predicts that in the absence of ART, the CD8 cytolytic killing rate is considerably less than the viral cytopathic death rate in all but one macaque. This result can explain the findings in Klatt et al. [5] and Wong et al. [6] of similar VL decline rates after ART initiation with or without prior CD8 depletion.

## Methods

### CD8 depletion experimental data

#### Experimental schedule

In Cartwright et al.[10], 13 rhesus macaques (RMs) were infected with SIVmac239. An ART regimen consisting of tenofovir, emtricitabine, raltegravir, and darunavir was started at week 8 after infection. One dose of the anti-CD8*α* depleting antibody, MT-807R1, was administered between 8–32 weeks after initiation of ART at 50 mg/kg intravenously. The experiment was designed to deplete CD8^+^ lymphocytes after viral loads were consistently undetectable or undetectable on at least three non-consecutive measurements. However, one animal never achieved undetectable viremia even after 32 weeks of ART. Animals were continued on ART and followed for another 8 weeks after CD8 depletion.

#### Viral load and CD8 data

SIV plasma viral load was measured longitudinally during the experiment. There were three different time phases during the experiment. Phase I is the pre-ART phase from week 0 to week 8, Phase II is from ART initiation to just before CD8 depletion, Phase III is from CD8 depletion to the end of experiment. In Phase III, all RMs have transient VL rebound following CD8 depletion, and VLs are controlled after CD8 T cell repopulation (S1 Fig). The sizes of peak rebound VL were different in different RMs. The VL data for all 13 RMs are shown in S1 Table and S2 Fig and the total CD8 T cell counts are shown in S2 Table and S3 Fig. For each monkey, their VLs and CD8 T cell counts are shown in S1 Fig. According to the criteria in Cartwright et al.[10], seven RMs that showed at least four consecutive time points with viremia below 60 copies/mL were “persistent suppressors”. We further classified these seven RMs into “fast controllers” (N = 3, S2 Fig) for those achieved viremia below the detection limit by 2 weeks after start of ART, and “intermediate controllers” (N = 4, S2 Fig) for those took longer to go below the detection limit. For the five RMs that showed a mix of undetectable and detectable levels (i.e., “intermittent suppressors” in Cartwright et al.[10]) as well as the one RM that never achieved undetectable viremia, we call them “slow controllers” (N = 6, S2 Fig).

### Parameter estimation

We built mathematical models to explain the VL data and infer mechanisms. We used the L-BFGS-B algorithm [11] in R to fit the models to the data by maximizing the log-likelihood function:

$$\arg\;\max_{\theta ,\sigma }\sum_{t}\left[-\ln(\sigma \sqrt{2}\pi )-0.5{\left(\frac{\log_{10}v(t)-\log_{10}f(\theta ,t)}{\sigma }\right)}^{2}+\ln\;\Phi (\frac{\log_{10}LOQ-\log_{10}f(\theta ,t)}{\sigma })-\ln\;\Phi (\frac{\log_{10}3.1-\log_{10}f(\theta ,t)}{\sigma })\right].$$

We assume *v*(*t*) = *f*(*θ*,*t*) + *e*, where *v*(*t*) is the measured viral load at time *t*, *θ* is a vector of parameters to estimate, *f*(*θ*,*t*) is the model simulated VL with parameters *θ* at time *t*, and *e* is the error between the theoretical model and the data, which we assume is normally distributed with variance *σ*^2^:*e*~*N*(0,*σ*^2^) [12, 13]. LOQ is the limit of quantification for censored VL data equal to 30 copies/mL [10]. We use Φ(*x*), the cumulative density function (CDF) of the Gaussian distribution to compute the probability that the model simulated VL *f*(*θ*,*t*) is below the LOQ. We assume that the residual plasma VL under therapy is not smaller than 3.1 copies/mL [14, 15], and incorporate this constraint in the log-likelihood function [16]. We run 100 data fittings for each RM with uniformly sampled initial parameter values from the log10-scaled parameter space. We select the parameter values from the best fits that maximize the log-likelihood. We compare models and parameter choices by calculating the Bayesian Information Criteria (BIC). In general, we prefer the parameter choices that give the smallest overall BIC. The typical guideline to accept one model over another is to have a BIC at least 2 points smaller. However, in our fitting, we preferentially accept the parameter choices that give the smallest BIC, regardless of whether the difference is larger or smaller than 2 points.

## Results

### Cytotoxic T Lymphocyte-Viral Control (CTL-VC) model

**Fig 1:**
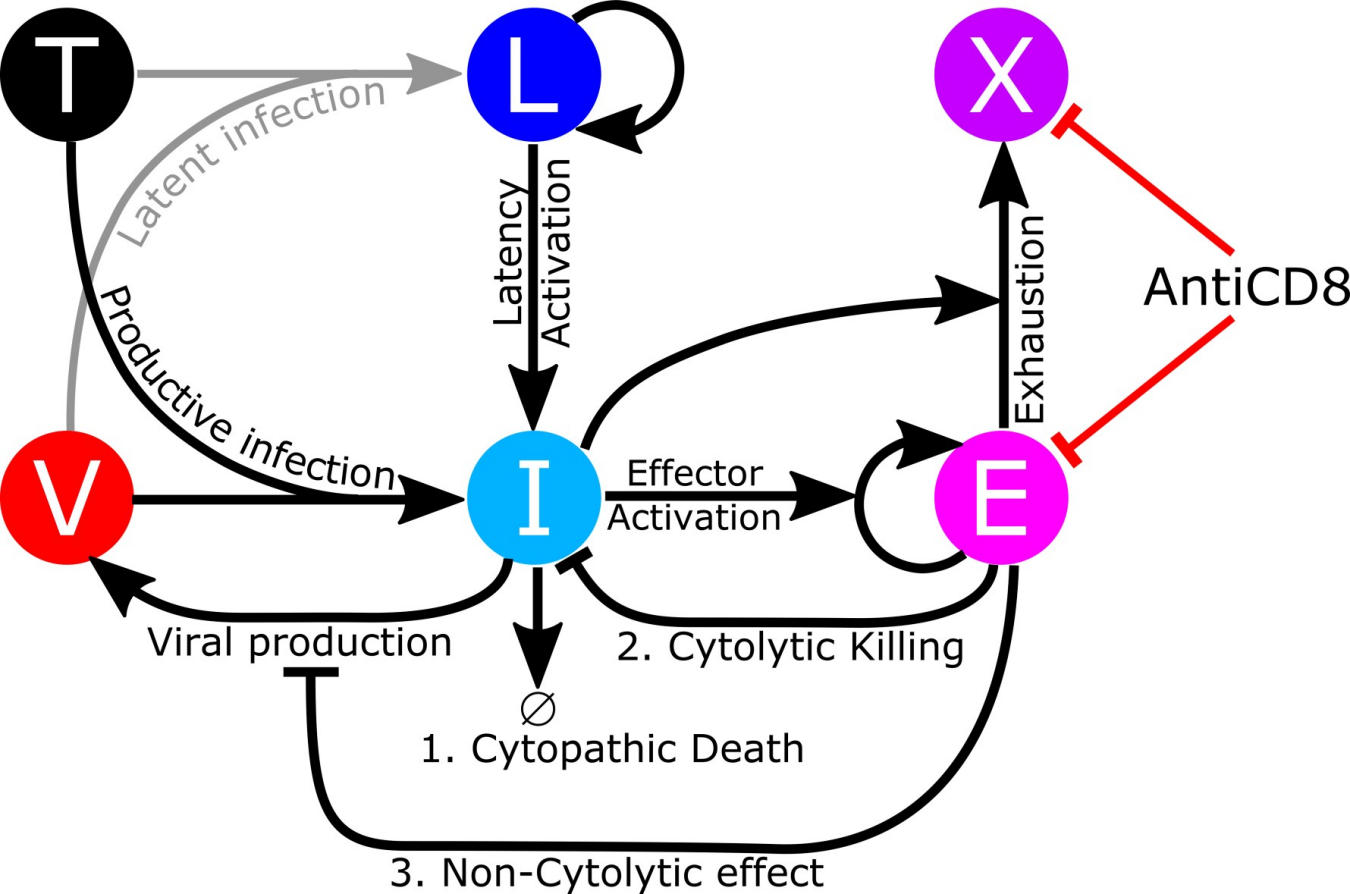
The cytotoxic T lymphocytes viral control (CTL-VC) model explicitly incorporates CD8 cytolytic killing and non-cytolytic effects on viral suppression, as well as antigen-induced CD8 T cell exhaustion. Virus (*V*) can infect target cells (*T*) through two pathways. A small fraction of the infection events generates latently infected cells (*L*) and the remaining fraction generate productively infected cells (*I*). Latently infected cells (*L*) can be activated into productively infected cells (*I*). Viruses are produced from productively infected cells (*I*). Latently infected cells (*L*) can proliferate. Productively infected cells (*I*) can die due to cytopathic effects or can be killed by SIV-specific CD8 cytolytic T lymphocytes (*E*) through cytolytic effects. CTL effector cells (*E*) can suppress the viral production from productively infected cells (*I*) through non-cytolytic effects. Productively infected cells (*I*) activate the effector cells (*E*) into clonal expansion and simultaneously exhaust the effector cells (*E*) into exhausted state (*X*). Anti-CD8 antibody deplete both effector and exhausted cells. The generation and death of target cells (*T*) and effector cells (*E*) and the death of exhausted cells (*X*) and the clearance of virions (*V*) are omitted from the diagram.

To understand the mechanism by which VL is controlled by CD8^+^ lymphocytes, we built a mathematical model to describe the dynamics of target cells (*T*), latently infected cells (*L*), productively infected cells (*I*), virus (*V*), effector cells (*E*) and allowed for the possibility that effector cells can become exhausted cells (*X*). As the anti-CD8*α* depleting antibody, MT-807R1, used in this study will deplete both CD8^+^ T cells and NK cells the effector cell population could comprise both cell types. As only CD8^+^ T cell levels were measured during the experiment, we focus on the CD8 T cell response and use the term cytotoxic T lymphocyte (CTL) interchangeably with the term effector cell with the understanding that the effector cell population could also contain NK cells. The model is a generalization of the model in Conway and Perelson [17], which was developed to explain the phenomenon of post-treatment control (PTC) observed in the VISCONTI study [18, 19]. The CTL-VC model is given by Eq 1 and illustrated in Fig 1.

$$\begin{array}{l} \frac{dT}{dt}=\lambda -d_{T}T-\left(1-\epsilon \right)\beta VT\\ \frac{dL}{dt}=\alpha_{L}\left(1-\epsilon \right)\beta VT+\left(\rho -a-d_{L}\right)L\\ \frac{dI}{dt}=\left(1-\alpha_{L}\right)\left(1-\epsilon \right)\beta VT-\delta I+aL-mEI\\ \frac{dV}{dt}=\frac{p}{1+\eta E}I-cV\\ \frac{dE}{dt}=\lambda_{E}+b_{E}\frac{I}{K_{B}+I}E-d_{E}\frac{I}{K_{D}+I}E-\mu E-k_{d}\frac{Ab\left(t\right)}{EC_{50}+Ab\left(t\right)}E\\ \frac{dX}{dt}=d_{E}\frac{I}{K_{D}+I}E-d_{X}X-k_{d}\frac{Ab\left(t\right)}{EC_{50}+Ab\left(t\right)}X \end{array}$$ Eq 1

Target cells (*T*) are constantly produced at rate *λ* and die at per capita rate *d*_*T*_. Virus (*V*) can infect target cells (*T*) at rate *β*. A fraction, *α*_*L*_, of the infection events generate latently infected cells (*L*) and the remaining fraction generate productively infected cells (*I*). Viral infection can be suppressed by ART with a drug efficacy, *ϵ*, where 0 < *ϵ* < 1. Latently infected cells (*L*) can be activated into productively infected cells (*I*) at a small rate *a*. Latently infected cells (*L*) proliferate at per capita rate *ρ* and die at per capita rate *d*_*L*_. Productively infected cells (*I*) die at per capita rate *δ* due to a combination of viral cytopathic effects, activation induced cell death and natural death, and are killed by SIV-specific effector cells (*E*) at per capita rate *mE*. Virus is produced from productively infected cells at a maximum rate *p* per cell, and are rapidly cleared at rate *c* per virion. CTL effector cells (*E*) can reduce the viral production rate *p* by the factor (1 + *ηE*) due to non-cytolytic effects [3, 20–23]. CTL effector cells (*E*) are produced at rate *λ*_*E*_ and die at per capita rate *μ*. Exhausted CD8 effector cells die at per capita rate *d*_*X*_. Also, the effector cells (*E*) are activated into clonal expansion by interacting with productively infected cells (*I*) according to a saturating term with a maximum growth rate *b*_*E*_ and 50% saturation coefficient *K*_*B*_. Simultaneously, the effector cells (*E*) become exhausted (*X*) according to a saturating term with maximum rate *d*_*E*_ and 50% saturation coefficient *K*_*D*_ ≫ *K*_*B*_. This model for effector cell expansion and exhaustion has been introduced previously by Conway and Perelson [17] and by Bonhoeffer et al. [24] and Adams et al. [25] to model effector cell expansion at low viral loads and “immune impairment” at high viral loads. To study how depleting the CD8^+^ lymphocytes affects the post-CD8 depletion viral rebound, we explicitly incorporate a CD8 depletion term in the model. Both effector and exhausted cells are depleted by the anti-CD8 antibody at per capita rate $k_{d}\frac{Ab(t)}{EC_{50}+Ab(t)}$, where *k*_*d*_ is the maximum depletion rate and *EC*_50_ is the 50% effective antibody concentration. All parameters and their descriptions and fixed values are listed in Table 1.

**Table 1:** Description of model parameters for the CTL-VC model.

Parameters	Values	Descriptions	References
**Viral dynamics**			
*λ*	10^4^ *cells mL*^−1^ *d*^−1^	Target cell production rate	[26]
*d*_*T*_	0.01 *d*^−1^	Target cell death rate	[27]
*β*	Estimated in the CTL-VC model by varying through values in 1.5 × 10^−8^,9.0 × 10^−8^]*mL d*^−1^	Mass-action infectivity, estimated in the range from 5.75 × 10^−10^ to 1.25 × 10^−7^ *mL d*^−1^ [28].	Estimated for the CTL-VC model.
*δ*	Estimated in the CTL-VC model by scanning through values between 0.2 and 0.5 *d*^−1^.	Infected cell cytopathic death rate	Estimated for the CTL-VC model.
*p*	[1000, 8000] *virions cells*^−1^ *d*^−1^ for the CTL-VC model.	Viral production rate.	Estimated for the CTL-VC model.
*η*	[10^−7^,10^2^] for the CTL-VC model.	Strength of the CD8 non-cytolytic effect.	Estimated
*c*	23 *d*^−1^	Viral clearance rate	[29]
*ϵ*	0.9	Drug efficacy	Fixed at 0.9 for the CTL-VC model.
**Latent cell dynamics**			
*t*_1/2_	38 days, *ρ* − *d*_*L*_ − *a* = −0.018 *d*^−1^	Latent reservoir half-life for animals under short-term ART, estimated from data in Dinoso et al. [30]. According to the measured frequencies of circulating resting CD4 T cells harboring replication-competent virus described on page 9251 in Dinoso et al. [30], we computed the decay slope for the latent reservoir under short-term ART as 0.018 *d*^−1^ corresponding to a half-life of 38 days.	[30]
*a*	0.002 *d*^−1^	Latent cell activation rate	[28]
*d*_*L*_	0.041 *d*^−1^	Latent cell death rate	
*ρ*	0.025 *d*^−1^	Latent cell proliferation rate	
*α*_*L*_	[10^−6^,10^−1^]	Probability of new infected cells becoming latent	Estimated
**Effector and exhausted cell dynamics**			
*m*	10^−4^ *mL cell*^−1^ *d*^−1^	Effector cell killing rate	Chosen to scale effector cell response
*λ*_*E*_	500 *cell mL*^−1^ *d*^−1^	Effector cell basal production rate	Chosen to scale effector cell response
*b*_*E*_	1.0 *cells mL*^−1^ *d*^−1^	Antigen induced maximum effector cell proliferation rate	Chosen to scale effector cell response
*d*_*E*_	[0.1, 20], *cells mL*^−1^ *d*^−1^	Antigen induced maximum effector cell exhaustion rate	Estimated
*μ*	0.75 *d*^−1^	Effector cell death rate	
*d*_*X*_	0.5 *μ*	Exhausted cell death rate	
*μ*_*T*_	0.011 *d*^−1^	Natural death rate of CD8 T cells	[27]
*K*_*B*_	[10^−3^, 20], *cells mL*^−1^	Saturation parameter for effector cell production	Estimated
*K*_*D*_	*K*_*D*_ = *f* × *K*_*B*_, with *f* estimated in the CTL-VC model by varying it through values in [30, 70] *cells mL*^−1^ with an interval of 5 *cells mL*^−1^.	Saturation coefficient of effector cell exhaustion	Estimated for the CTL-VC model.
**CD8 depletion**			
*k*_*d*_	[0.1, 20], *d*^−1^	Maximum depletion rate	Estimated
*EC*_50_	Estimated by varying through values in [0.0001, 0.003] *μg mL*^−1^	Effective concentration for 50% maximum depletion	Estimated
**Anti-CD8 antibody PK parameters**			
*c*_1_	200 *μg mL*^−1^	Concentration parameter for the first phase	Calculated from the dose of the antibody
*c*_2_	700 *μg mL*^−1^	Concentration parameter for the second phase	
*k*_1_	[0.001, 5], *d*^−1^	Decay rate in the first phase	Estimated
*k*_2_	[0.001, 5], *d*^−1^	Decay rate in the second phase	Estimated

### Antibody decay parameters

The pharmacokinetics of the CD8 depleting antibody MT-807R1 have not been characterized. Here we assume that after infusion the Ab decays according to a biphasic model: $Ab(t)=c_{1}\;e^{-k_{1}t}+c_{2}e^{-k_{2}t}$, where *t* is the time after antibody administration. We estimated the antibody decay parameters for each RM by fitting a phenomenological model to the dynamics of the total CD8 T cell data during depletion. We assume the dynamics of the total CD8 T cells follow the equation: $\frac{dTot}{dt}=\lambda_{T}-\mu_{T}Tot+k_{p}Tot(1-\frac{Tot}{Tot^{ss}})-k_{d}\frac{Ab(t)}{EC_{50}+Ab(t)}Tot$, and assume the total CD8 T cell population is at steady state before depletion. Note that the total CD8 population is not the same as *X*+*E*, which is the total antigen-specific CD8 population. We use the total CD8 T cell count data before depletion as its steady state value *Tot*^*ss*^ and we use a logistic term to model the homeostatic control of total CD8 T cells. We assume the maximum proliferation rate of CD8 T cells under lymphopenic conditions is *k*_*p*_ = 6 *d*^−1^ [31]. We fix *μ*_*T*_ = 0.011 *d*^−1^ [27] (Table 1), then we choose *λ*_*T*_ = *μ*_*T*_*Tot*^*ss*^ for each RM to maintain the steady state CD8 T cell level. The dose of anti-CD8 antibody MT-807R1 in the experiment was 50 mg/kg [10]. Assuming an average blood volume of 55mL/kg for RMs [32], we calculate the maximum antibody concentration *C*_*max*_ = 50/55 mg/mL≈900 *μ*g/mL. We let *c*_1_ = 200 *μ*g/ml and *c*_2_ = 700 *μ*g/ml to have *c*_1_ + *c*_2_ = *C*_*max*_. We estimate the values of *k*_*d*_, *k*_1_, and *k*_2_ that lead to the best fit of the model for the total CD8 T cell dynamics to the CD8 data for each animal, while scanning *EC*_50_ through the values 0.0001 *μg*/*mL*, 0.0005 *μg*/*mL*, 0.001 *μg*/*mL*, 0.002 *μg*/*mL*, and 0.003 *μg*/*mL*. We fixed *EC*_50_ = 0.001 *μg*/*mL* as it gave the smallest overall BIC. The fits to the total CD8 T cell dynamics with *EC*_50_ = 0.001 *μg*/*mL* in all RMs are shown in S4 Fig, and the estimated parameters for anti-CD8 antibody are listed in S3 Table. Note that due to lack of pharmacokinetic data, the antibody decay parameters estimated from this fitting are only for simulating the phenomenological dynamics of CD8 T cell depletion. They do not necessarily reflect the realistic *in vivo* parameter values.

### Data fitting and estimated parameters

We estimate 5 model parameters by fitting the CTL-VC model to the VL data (Table 1), namely the probability of latent infection *α*_*L*_, the viral production rate *p*, the antigen induced maximum effector cell exhaustion rate *d*_*E*_, the 50% saturation coefficients for effector cell production *K*_*B*_, and the strength of the CD8 non-cytolytic effect *η* that modulates the viral production rate, while scanning *δ* through different values between 0.15 *d*^−1^ and 0.90 *d*^−1^ with an interval of 0.05 *d*^−1^ and scanning *β* through values between 1.5 × 10^−8^ *mL d*^−1^ and 4.5 × 10^−8^ *mL d*^−1^ with an interval of 5 × 10^−9^ *mL d*^−1^ with the understanding that if the best-fit values of these parameters occur at the ends on these intervals we would extend the searched parameter interval. The upper end of search interval for *δ* was chosen to be less than 1.5 *d*^−1^, a value estimated by Brandin et al. [33] using a model without effector cells, so as to allow effector cell killing to play a role. We fit the value of *α*_*L*_ so as to allow the possibility that each RM has a different latent reservoir size. Because CD8 T cell exhaustion occurs later in infection than activation, we assume the 50% saturation coefficient for exhaustion *K*_*D*_ is larger than *K*_*B*_. Here we let *K*_*D*_ = *f* × *K*_*B*_, and vary *f* through different values between 30 and 70 with an interval of 5. The value of *f* = 55 gives the smallest overall BIC. We therefore fix *K*_*D*_ = 55 *K*_*B*_ in all our subsequent analysis. We fit the CTL-VC model to the data and compute the overall BIC from the best-fits for each different value of *δ* and *β* in each animal (S4 Table and S5 Table). The values of *δ* = 0.40 *d*^−1^ and *β* = 3.0 × 10^−8^ *mL d*^−1^ gives the smallest total BIC. Although the best-fit values for *δ* and *β* are different among animals in terms of BIC, the best BICs of most animals are within 2 of the BIC obtained using *δ* = 0.40 *d*^−1^ and *β* = 3.0 × 10^−8^ *mL d*^−1^, and thus for simplification we fix *δ* = 0.40 *d*^−1^ and *β* = 3.0 × 10^−8^ *mL d*^−1^. All other fixed parameter values are listed in Table 1.

**Fig 2:**
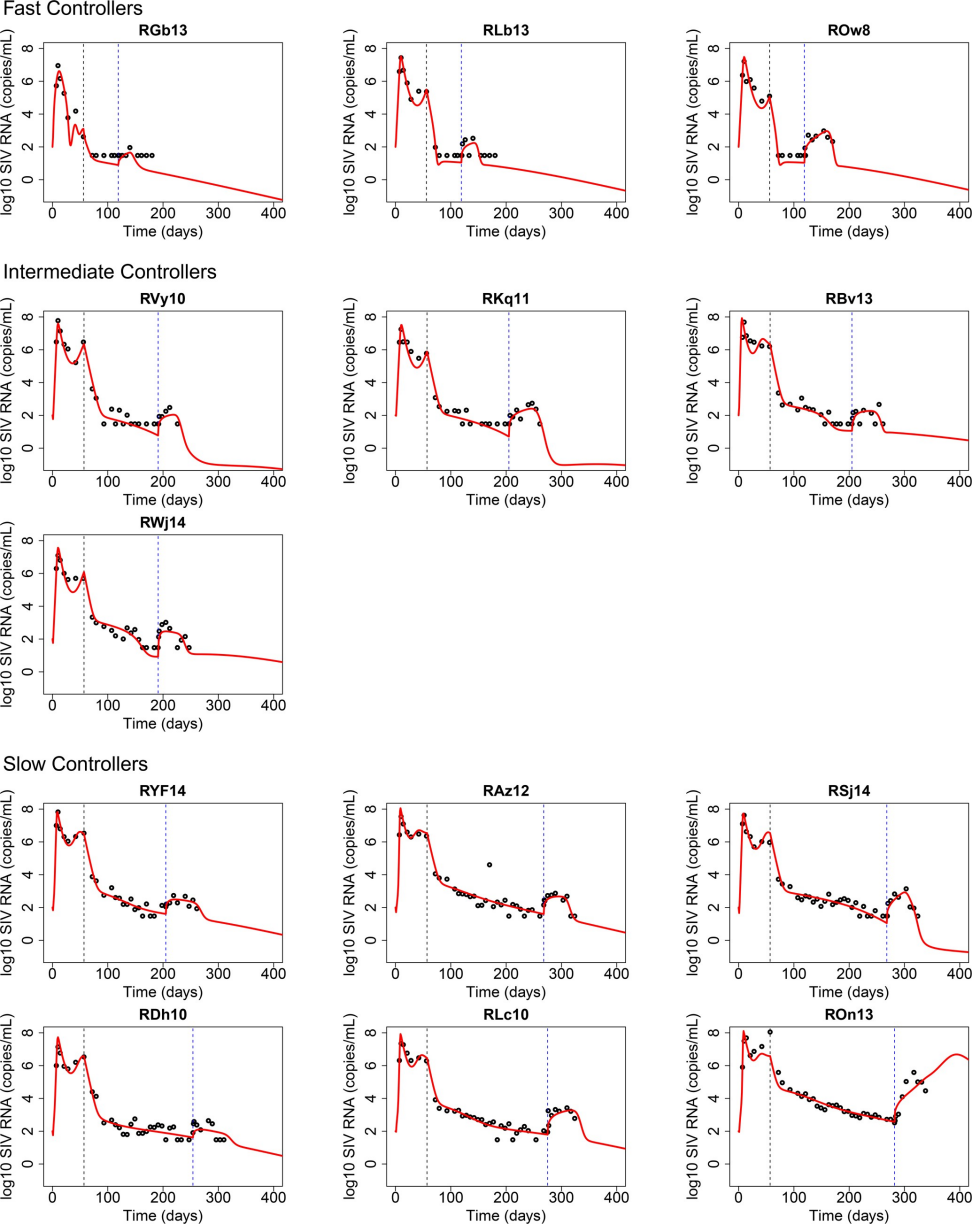
Fits of the CTL-VC model (red lines) to the VL data (black dots). The first vertical dashed line indicates the start time of ART, and the second vertical dashed line indicates the start time of anti-CD8 antibody.

Estimated parameters are listed in Table 2. The estimated values for *α*_*L*_ and *p* are within ranges estimated in other studies [28, 34]. The best-fit of the CTL-VC model to the data accurately captured the VL dynamics in all three phases, pre-ART, post-ART, and post-CD8 depletion in most RMs (red lines in Fig 2). The model correctly captured the first VL peak during primary infection in all RMs, and the slow second phase decay in VL dynamics during ART. The model also correctly captured the VL dynamics after ART and before CD8 depletion. Importantly, the model correctly captured the post-CD8 depletion VL rebound in all RMs, including the small post-depletion VL peak in RGb13, which has only one supporting data point above the detection limit (Fig 2).

**Table 2:** Estimated parameter values for the CTL-VC model.

**RM**	*α*_*L*_	*p* (*virions cell*^−1^*d*^−1^)	*d*_*E*_ (*cells mL*^−1^*d*^−1^)	*K*_*B*_ (*cells mL*^−1^)	*η*	*σ*	−*LL*
****RGb13****	7.79E-05	4010	0.37	1.40E+01	9.38E-04	0.35	10.20
****RLb13****	2.79E-04	4622	0.78	2.78E-01	1.71E-03	0.35	10.40
****ROw8****	3.32E-04	4852	0.75	2.58E-01	1.90E-03	0.37	10.15
****RVy10****	6.58E-04	5201	2.71	5.67E-03	9.22E-03	0.40	16.71
****RKq11****	1.05E-03	4567	1.58	4.95E-03	4.65E-03	0.39	16.56
****RBv13****	1.43E-03	3204	1.18	4.01E-02	4.45E-05	0.48	23.66
****RWj14****	9.13E-03	2052	0.93	1.17E-01	1.58E-04	0.41	17.02
****RYF14****	5.10E-03	2969	7.25	9.55E-01	1.78E-03	0.27	7.10
****RAz12****	1.19E-02	4209	44.10	9.97E+00	5.59E-03	0.45	28.27
****RSj14****	3.62E-03	5891	4.17	1.53E-02	1.23E-02	0.30	12.49
****RDh10****	6.28E-03	2353	6.43	2.46E+00	8.63E-04	0.42	25.80
****RLc10****	1.99E-02	3701	6.22	3.08E+00	2.60E-03	0.32	14.56
****ROn13****	9.97E-02	4972	29.48	5.00E+01	4.57E-03	0.42	23.88

Based on simulations using the estimated parameters, the model predicted that the VL will not rebound if CD8 T cells are not depleted (S5 Fig). Furthermore, the model predicted that if these RMs are CD8 depleted in the absence of treatment, a transient increase in viremia will occur with different amplitudes in different animals (S6 Fig), as observed in previous experiments [4]. Interestingly, the model also predicted the total CD4^+^ T cell dynamics well without fitting to the CD4^+^ T cell data (S7 Fig).

### The latent reservoir has a more significant contribution to the post-depletion peak VL than drug efficacy

**Fig 3:**
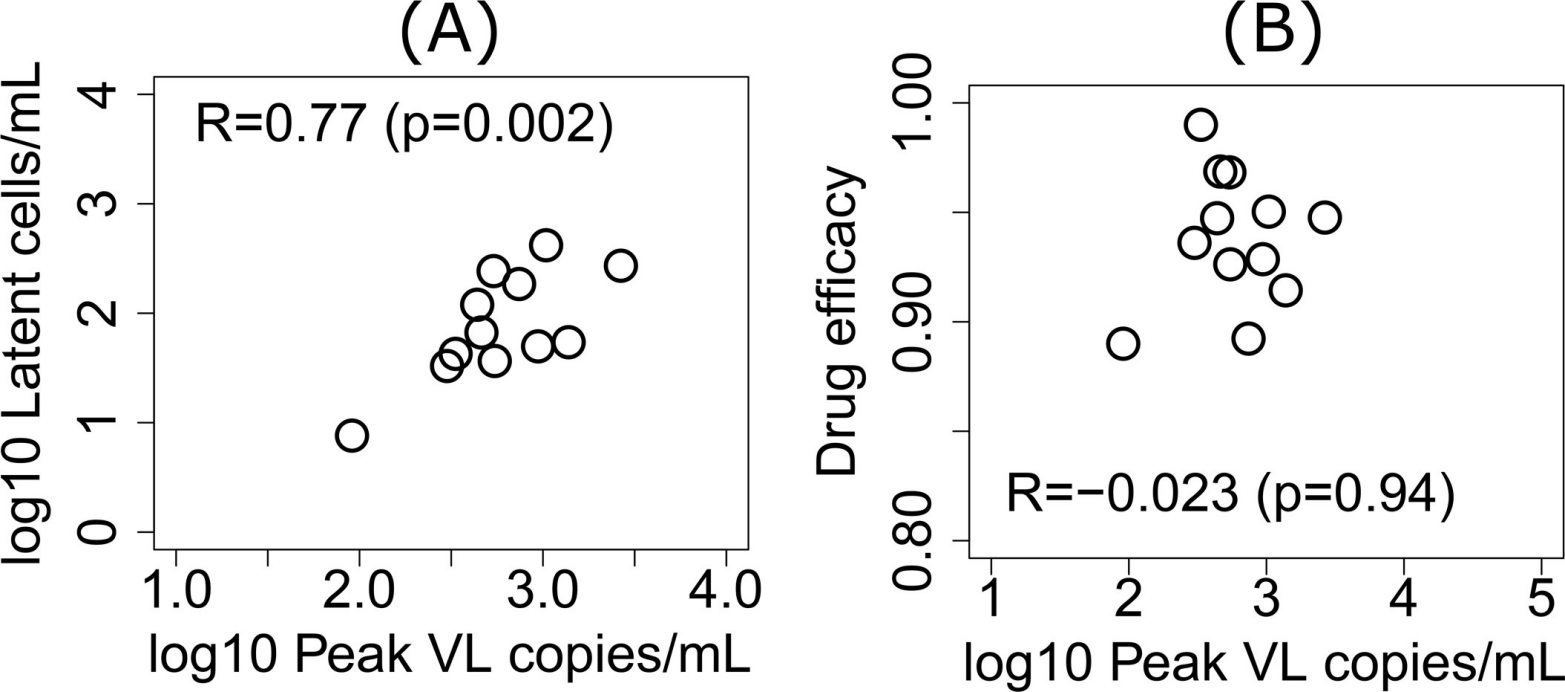
The CTL-VC model predicted a strong positive correlation (A) between the peak VL after CD8 depletion and the predicted latent reservoir size before depletion, when drug efficacy was fixed at 0.9, and a weaker negative correlation (B) between the post depletion peak VL and the estimated drug efficacy when the fraction of latent infection *α*_*L*_ was fixed at different values for each animal.

The size of the post-CD8-depletion VL peak is different among RMs (S2 Fig). We hypothesize that the size of latent reservoir and drug efficacy might be contributing to the size of the post-CD8 depletion VL peak. A strong correlation (*R* = 0.6) was demonstrated between post-depletion peak VL and the latent reservoir size measured by SIVgag DNA copy number in Cartwright et al. [10]. Further, longitudinal single genome sequencing of plasma virus showed that the sequences of the post-CD8 depletion viruses were more similar to sequences derived at peak viremia than to those derived immediately prior to ART suggesting that the rebound virus may have been archived in long-lived or latently infected cells [10]. To test the hypothesis that latent reservoir size influences the post-CD8-depletion VL peak, we first calculated the correlation between the peak VL in the data and the pre-CD8-depletion latent reservoir size predicted by the CTL-VC model using the estimated parameter values (Table 2). The peak VL after CD8 depletion has a strong positive correlation with the predicted pre-depletion latent reservoir size (*R* = 0.77 with *p* = 0.002) (Fig 3A). These results suggest that the latent reservoir significantly contributes to the viral rebound after CD8 depletion.

To compare the relative contributions of drug efficacy and latent reservoir size to the peak VL after CD8 depletion, rather than fixing the drug efficacy (Table 1), we estimate the drug efficacy for each RM by fitting the CTL-VC model to the VL data. To maintain the number of parameters we fit at 5, rather than fitting the probability of infection yielding a latently infected cell, *α*_*L*_, we now allow *α*_*L*_ to take a fixed value 10^−6^,10^−5^,10^−4^,10^−3^,10^−2^, or 10^−1^ that yields the best-fit each different animal. The estimated parameters along with the value of *α*_*L*_ for each animal are listed in S6 Table. Based on the estimated drug efficacy, the post-depletion peak VL has a weak and not statistically significant negative correlation with drug efficacy *ϵ* (*R* = −0.023 with *p* = 0.94) (Fig 3B). This result confirms that contribution of the latent reservoir to the size of the peak VL after CD8 depletion is more significant than the contribution from drug efficacy and is consistent with the sequencing data in Cartwright et al. [10] indicating that the virus emerging after CD8 depletion is similar to “peak” virus and not the virus circulating immediately prior to ART initiation.

### SIV-specific CD8 T cells contributed to viral control

**Fig 4:**
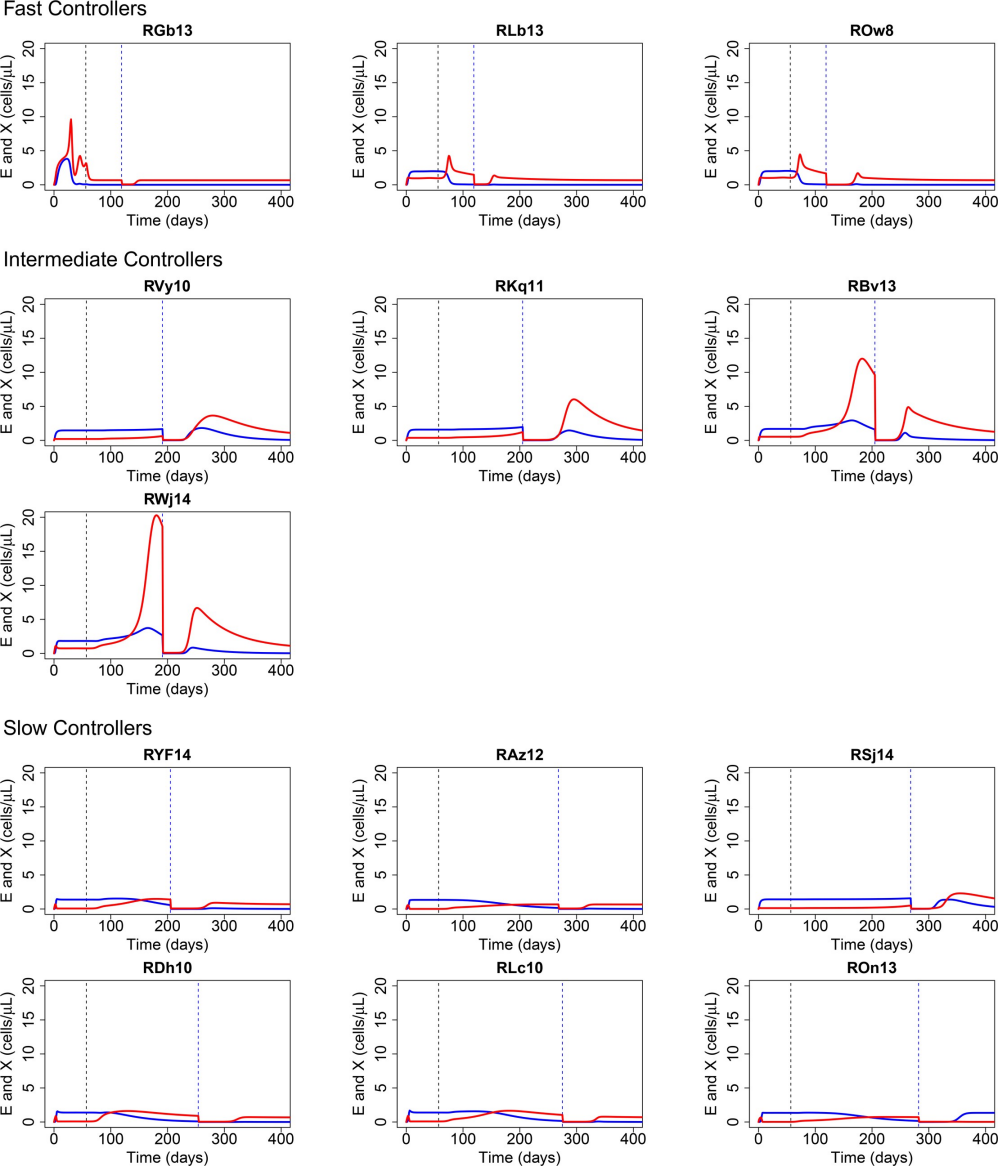
Simulations of effector cell (red lines) and exhausted cell (blue lines) dynamics in the CTL-VC model. The first vertical dashed line indicates the start time of ART, and the second vertical dashed line indicates the time of anti-CD8 antibody administration.

To understand the role of SIV-specific effector CD8 T cells in controlling viral infection, we simulated the trajectory of the effector cell (*E*) population and the exhausted cell (*X*) population based on the estimated parameters. In most RMs, CD8 T cells show significant exhaustion during primary infection before ART resulting in an exhausted population that is larger than the effector cell population (Fig 4). Early exhaustion of CD8 memory T cells has also been seen during acute viral infections of mice with lymphocytic choriomeningitis virus [35]. Interestingly, after initiation of ART, our model predicted an increase in the effector cell population (red lines in Fig 4) in most RMs. As a result, the effector cell population become dominant in most animals after initiation of ART (Fig 4). We also predicted that the total SIV-specific CD8 T cells (S8 Fig) including both effector and exhausted cells hardly changes in most animals, with the exception of two intermediate controller animals in which there is a predicted increase (see S2 Fig). Why these animals show a predicted increase is not clear, although increases in antigen specific CD8 T cells after ART initiation have been previously observed but could be influenced by factors such as redistribution, which are not part of our model.

### CD8 T cell cytolytic killing plays a more important role than SIV-induced cytopathic death during ART

**Fig 5:**
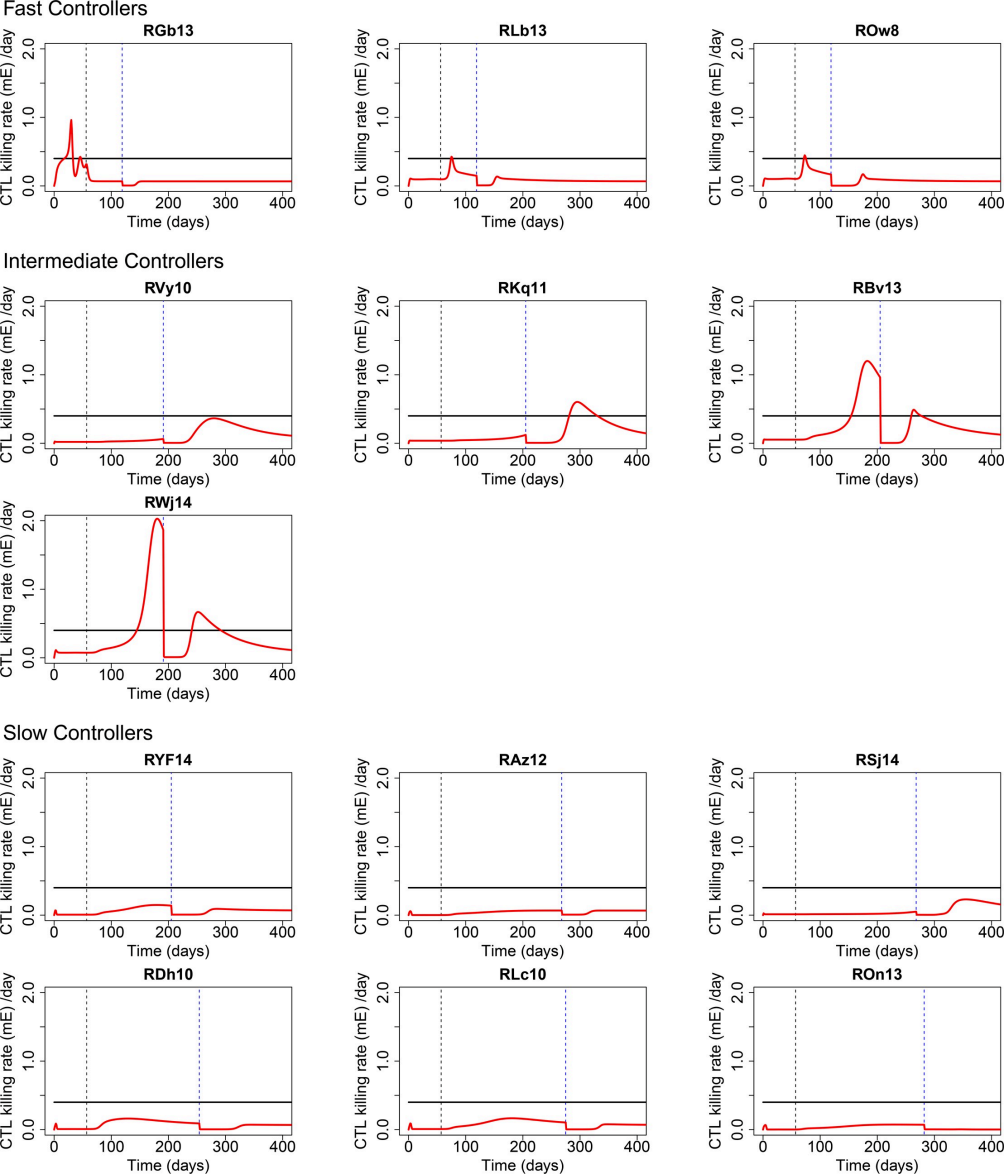
Predicted CD8 cytolytic killing rates, *mE*, (red lines) from the CTL-VC model. The black horizontal lines indicate the fixed cytopathic death rate of infected cells, *δ* = 0.40 *d*^−1^.

HIV/SIV infected cells can die by viral cytopathic effects, activation induced cell death, natural death or by CD8 T cell cytolytic killing. Based on the parameters estimated from our modeling, we compared the predicted dynamics of the CD8 cytolytic killing rate, *mE*, with the rate of cell death induced by viral cytopathic effects *δ* (Fig 5). In phase I, before ART, the cytolytic killing rate tends to be small (Fig 5). However, after ART is started, the cytolytic killing rate increases in some RMs due to the expansion of the CD8 effector cell population (Fig 4). The peak cytolytic killing rate during ART can be as high as about 2.0 *d*^−1^ (*e*.*g*. RWj14 in Fig 5), which is significantly higher than the cytopathic cell death rate (*δ* = 0.40 *d*^−1^). According to the model, when VL control is achieved, the CD8 cytolytic killing (*mE*) starts to decline (S9 Fig). In most RMs, the cytolytic killing rate declines back to a level smaller than the cytopathic death rate.

Overall, these results suggest an important role of CD8 T cell cytolytic killing in control of the viral load during ART. Our modeling suggests that the cytolytic killing rate of CD8 T cells is not constant, rather it is dynamically changing as the effector cell population size is changing, and can be dramatically affected by the residual VL. In comparison with the death rate of productively infected cells due to cytopathic effects (black horizontal lines in Fig 5), the cytolytic killing rate in some animals can be significantly larger during ART.

### CD8 T cell cytolytic and non-cytolytic effects both contribute to viral suppression

To compare the role of CD8 cytolytic killing and non-cytolytic effects on suppressing viral replication, we further fit the CTL-VC model to the VL data with either no non-cytolytic effect (*η* = 0) or with no cytolytic killing (*m* = 0). We then compared the qualities of the fits with the original CTL-VC model, which has both cytolytic and non-cytolytic effects. Estimated parameters for both mechanisms are listed in S7 Table and S8 Table, respectively. The fitting qualities of the CTL-VC model with either only cytolytic effect or only non-cytolytic effect are worse than that of original CTL-VC model with both effects, while the fitting quality of CTL-VC model with only cytolytic effect is the worst.

To further study the role of non-cytolytic effect in viral suppression, we plotted the predicted viral production rate *p*/(1 + *ηE*) based on the estimated parameters of CTL-VC model (Table 2) for all 13 RMs (red lines in S10 Fig). In some animals, the maximum suppression of viral production can be more than 50% (S10 Fig), while the effect can be minor in other animals. In the model with no CD8 cytolytic killing (*m* = 0), the viral production rates are predicted to be suppressed to close to 0 *d*^−1^ in some animals through the non-cytolytic effect (S11 Fig). This overly strong suppression of viral production (S11 Fig) does not seem biologically realistic and suggests that CD8 non-cytolytic effects might not be the only mechanism to suppress viral replication. Overall, the comparisons of fitting qualities between two effects suggest that CD8 cytolytic and non-cytolytic effects likely are both important for viral suppression, but quantitative comparisons are difficult.

### Second phase of VL decay

One interesting feature of the CTL-VC model is that it captures the slow kinetics of the second phase of viral decay without introducing a population of long-lived infected cells. The viral load data in most intermediate controllers and slow controllers exhibits a fast first phase decay and a slow second phase decay (S12 Fig), while in the fast controllers there is not enough data to characterize the second phase. The half-life of second phase decay in slow controllers (~36.3 days on average) is longer than in intermediate controllers (~22 days on average) (S12 Fig). Interestingly, according to our model the slow controllers have smaller CD8 cytolytic killing rates than those intermediate controllers (Fig 5 and S9 Fig). This suggests that the dynamically changing contribution of effector cell killing could be responsible for the second phase of VL decay, an idea previously suggested in a modeling study by Arnaout et al [36].

To examine the effect of CD8 T cells on the half-life of second phase decay, we first compared the contributions of the two infected cell populations, *i*.*e*. latently infected cells and productively infected cells to the overall VL dynamics. To do this, we simulated the contributions to the VL of the two cell populations (S13 Fig) based on the estimated parameters from the CTL-VC model. Results showed that productively infected cells dominantly contributed to the VL during the initial phase of VL decay after initiation of ART (S13 Fig red lines). The second phase VL decay was mostly contributed by activation from latently infected cells (S13 Fig green lines). The calculated half-lives of second phase decay from simulations are consistent with those seen in the data. We further simulated VL dynamics using the estimated parameters but varying the CD8 effector cell killing rate, *m*, and found that strong CD8 T cell responses can reduce the half-life of second phase decay, while weak CD8 responses prolong it (S14 Fig).

To study whether these conclusions hold in a model with long-lived infected cells, we developed a new model, called the *CTL-VC long-lived infected cell* model, by explicitly incorporating the dynamics of infected long-lived cells (M*) and their contributions to viral production into the original CTL-VC model. We fit the *CTL-VC long-lived infected cell model* to the VL data and estimated the same 5 parameters as in the original CTL-VC model, while varying the density of uninfected long-lived cells *M*_0_, the infection rate *β*_*M*_, the cytopathic death rate *δ*_*M*_ and cytolytic killing rate *m*_*M*_ for long-lived cells over a large range. Estimated parameters are listed in S9 Table and the data fits are shown in S15 Fig. Details for the *CTL-VC long-lived infected cell model* are included in SI. The overall quality of fits with the *CTL-VC long-lived infected cell model* is slightly improved (total BIC = 689, S15 Fig) over that of the original CTL-VC model (total BIC = 703).

Simulations of the *CTL-VC long-lived infected cell model* using best-fit parameters suggest that the long-lived cell population does not significantly contribute to the second phase decay dynamics, which is mostly contributed by activation of latently infected cells (S16 Fig green lines). The long-lived infected cells contributed mostly to the VL during the transition from the first to the second phase decay (S16 Fig).

The positive correlation between the pre-depletion latent reservoir size and post-depletion peak VL remains strong in the *CTL-VC long-lived infected cell model* (*R* = 0.74 and *p* = 0.004) (S17A Fig). While the model also predicted a positive correlation between the pre-depletion long-lived cell population size and the post-depletion peak VL (*R* = 0.64 and *p* = 0.018) (S17B Fig), the magnitude of VL contribution from long-lived cell population is about 2–3 logs smaller than the contribution from latency activation. Furthermore, the predicted CD8 cytolytic killing rates for both productively infected (*mE*) and long-lived (*m*_*M*_*E*) infected cells of the CTL-VC *long-lived infected cell model* appears to have similar patterns as those of the original CTL-VC model (S18 Fig), *i*.*e*. *mE* increases after the start of ART and CD8 cytolytic killing plays an important role in some animals during ART. Overall, results from the *CTL-VC long-lived infected cell model* are compatible with the conclusions based on the original CTL-VC model.

### CD8 depletion before ART

Two groups studied the effect of CD8 T cells on VL decay by depleting CD8 T cells before ART in SIV-infected RMs [5, 6]. Both Klatt et al. [5] and Wong et al. [6] found that when CD8 T cells were depleted about one week before the initiation of ART there was no significant difference between the VL decline slope in these animals and in a control group with no CD8 depletion after ART was initiated. Based on these experiments, it was argued that the contribution of CD8 killing to suppressing viral replication in productively infected cells might be negligible in the setting of high virus production. However, the experiments were compatible with possible CD8 killing of cells before viral production began or with an effect of the eclipse phase in determining the viral decay slope [5, 6, 8].

**Fig 6:**
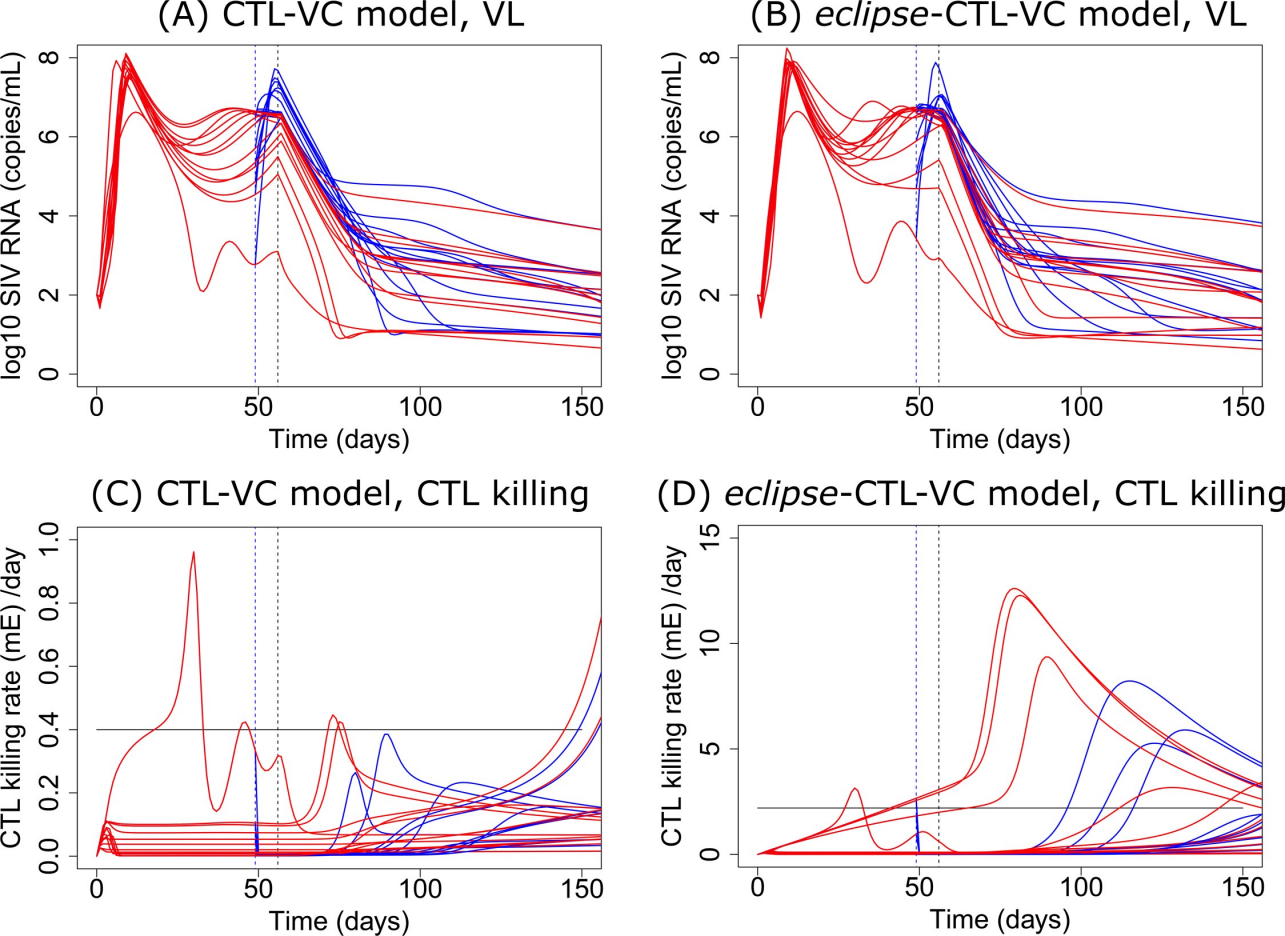
Depleting CD8 T cells one week before ART does not affect the first phase VL decay slope in most RMs due to small CTL killing rates. The first vertical dashed line indicates the time of CD8 depletion, and the second vertical dashed line indicates the start time of ART. Red lines show model predictions without CD8 depletion, and blue lines show predictions when CD8^+^ lymphocytes are depleted one week before ART. (A) Predicted viral dynamics from the CTL-VC model. (B) Predicted viral dynamics from the *eclipse*-CTL-VC model. (C) Predicted CTL killing rate (*mE*) from the CTL-VC model. (D) Predicted CTL killing rate (*mE*) from the *eclipse*-CTL-VC model. Black solid lines in (C) and (D) show the estimated cytopathic cell death rates in both models. See S2 Text for an explanation of why the estimated cytopathic killing rate δ = 2.20 *d*^−1^ is significantly higher than the death rate in the CTL-VC model. Both models showed the same VL decline slopes with or without CD8 depletion before ART, as well as smaller CTL killing rates than the cytopathic cell death rate in most RMs before ART.

To test if our model can also explain the phenomena of similar post-ART first phase VL decline slopes with or without CD8 depletion, we simulated the VL dynamics of all 13 RMs when their CD8 T cells are depleted one week before ART initiation based on the estimated parameters from the CTL-VC model. After CD8 T cells were depleted but before ART was started, most RMs had an average 1 log of VL increase (blue lines in Fig 6A). After the start of ART, the VL decline slope in most simulations with CD8 depletion (mean slope −0.33 *d*^−1^, blue lines in Fig 6A) are close to the decline slopes in simulations with no CD8 depletion (mean slope −0.35 *d*^−1^, red lines in Fig 6A), is consistent with the experimental observations in Klatt et al. [5] and Wong et al. [6]. According to our model, this is explained by the small contribution of CD8 cytolytic killing before the initiation of ART (Fig 6C) when VLs are high and most CD8^+^ T cells are exhausted (Fig 4). Thus, depleting CD8 T cells has little predicted effect. However, based on our modeling, the SIV-specific CD8 response reaches peak cytolytic killing rate around the time the VL becomes undetectable during ART. Therefore, depletion of CD8 T cells after VL control can have a more significant effect on VL rebound, as shown in Cartwright et al. [10].

We also tested the effect of infected cell killing during the eclipse phase on the first phase decline rate. To do this, we expanded the CTL-VC model by explicitly incorporating the eclipse phase of SIV infection (see the *eclipse*-CTL-VC model in SI for more details). Results show that the *eclipse*-CTL-VC model can also fit the data well (S19 Fig, estimated parameters in S10 Table) with a slightly worse BIC (total BIC = 727) than that of the original CTL-VC model (total BIC = 703). The results from the *eclipse*-CTL-VC model are consistent with the results from the CTL-VC model (Fig 6B), in that both models showed a VL increase and similar first phase VL decline slope after ART initiation when CD8 T cells are depleted about one week before ART (Fig 6). The *eclipse*-CTL-VC model also predicted the small contribution of CD8 cytolytic killing before the initiation of ART (Fig 6D), consistent with the CTL-VC model.

## Discussion

Cartwright et al. showed that CD8 T cells play important roles in controlling SIV infection even when animals are treated with ART [10] by observing that depleting CD8 T cells in ART-treated SIV-infected rhesus macaques (RM) was followed by viral resurgence with viral control being re-established after CD8 T cell repopulation. To study the detailed role of CD8 T cells in viral control, we built mathematical models and fit the models to the viral load data from this study.

To understand the detailed mechanism of viral control driven by CD8 T cells, we first built a viral kinetic model that explicitly incorporated latently infected cells as well as the dynamics of antigen-specific CD8 effector cells and CD8 exhausted cells, namely the Cytotoxic T Lymphocyte-Viral Control model (CTL-VC model). The CTL-VC model incorporated both CD8 cytolytic killing and non-cytolytic viral suppression. One important limitation of our work is that no killing marker or exhaustion marker was measured in the experiment and thus we have no direct experimental evidence of CTL activity or CD8 cell exhaustion, although as we showed in Results and discuss below the model assuming CTL activity and CD8 exhaustion is consistent with the observed viral dynamics. We further assumed the CD8 depleting antibody depleted both effector and exhausted cells. We constructed a simple antibody pharmacokinetics model and fit the model to the total CD8 T cell count data so that we could accurately represent the effects of the anti-CD8 antibody. We incorporated this into the CTL-VC model and then fit the VL data and estimated 5 model parameters. The CTL-VC model accurately captured the viral dynamics in all RMs (Fig 2).

Based on the model fits and the estimated parameters, we examined the contributions of the latent reservoir and drug efficacy to the peak value of the VL rebound after CD8 depletion. We found a strong correlation (*R* = 0.77 with *p* = 0.002) between the predicted latent reservoir size before CD8 depletion and the size of the peak VL after CD8 depletion. The estimated drug efficacies in different RMs showed a weak and not statistically significant negative correlation with the post-depletion peak VL (*R* = −0.023 with *p* = 0.94). This result suggests that the size of the latent reservoir or the factors that control the activation of latently infected cells are major contributors to the post CD8 depletion VL resurgence, consistent with the experimental data in Cartwright et al. [10] showing a correlation between SIV DNA^+^ cells and the post CD8 depletion VL peak and that the viral sequences that emerged post CD8-depletion were more similar to those derived at peak viremia than to those present immediately before ART was initiated.

We further examined the detailed roles of CD8 T cells during viral control in terms of effector activation and exhaustion. The predicted dynamics of the effector cell population is different among RMs, but most RMs were predicted to exhibit an increase in the SIV-specific CD8 T effector cell population during first 2–20 weeks after the initiation of ART, followed by a long-term decay (Fig 4). This result agrees with previous experimental observations that HIV-specific CD8^+^ T cells decay in the long-term (>20 weeks) during ART [37–39], but increase in the short-term after initiation of ART [38–41]. The model predicted that after initiation of ART, the effector cell population expands faster than the exhausted cell population in most RMs. As a result, the effector cell population becomes dominant in most RMs after a certain length of ART, which further suggests a significant role of SIV-specific CD8 T cells in viral control.

The relative contributions of CD8 cytolytic killing, non-cytolytic effects, and viral cytopathic cell death in viral control remain important undetermined quantities. Based on estimated parameters, our model suggests that the CD8 cytolytic killing rate dynamically changes with the VL. As CD8 exhaustion increases when the VL is high, the cytolytic killing rate becomes low. On the other hand, when the VL is low, loss of effector cells due to exhaustion occurs at a very low rate, and the CD8 effector cell population increases, as a result the cytolytic killing rate becomes elevated. Before the initiation of ART or without ART, most RMs have a high VL, which we predict results in a smaller cytolytic killing rate than the viral cytopathic-induced cell death in most RMs. At this stage, viral cytopathic effects are the major mechanism that causes infected cell death. Specifically, the model predicted that in the absence of ART, the CD8^+^ cytolytic killing rate is considerably less than the viral cytopathic death rate in all but one macaque.

However, after initiation of ART, the CD8 cytolytic killing rate starts to increase in most RMs due to expansion of the SIV-specific CD8 effector cell population with little exhaustion. The CD8 cytolytic killing rate reaches its peak rate (as high as 2.0 *d*^−1^ in some RMs) when the VL is very low under ART, which is somewhat surprising since the stimulus for CD8 expansion is also low. During the second phase of VL decay under ART, when the VL is low, cell death is mainly due to CD8 cytolytic killing rather than cytopathic death in some animals. Overall, our model suggests a dynamic role of CD8 cytolytic killing. In comparison with the cytopathic effect, CD8 cytolytic killing might be playing a minor role before ART or with no ART if viral loads are high which leads to CD8 exhaustion. Conversely, cytolytic killing can play an important role during VL suppression under ART if CD8 exhaustion is low. In addition, we studied the role of the CD8 non-cytolytic effect in control of viral infection by comparing data fitting qualities of different models. Our modeling suggests both cytolytic and non-cytolytic effects are important during viral control. However, given the data we have it is difficult to quantitatively compare the relative contributions of the CD8 non-cytolytic effect and cytolytic effect on viral control.

RMs in the experiment by Cartwright et al. can be grouped into 3 groups: fast, intermediate and slow controllers, by their different rates to reach VL control after start of ART [10] (S12 Fig). Interestingly, the mean half-life of second phase VL decay in slow controllers is longer than that of intermediate controllers (S12 Fig). According to our model the slow controllers have smaller CD8 cytolytic killing rates than those intermediate controllers (Fig 5 and S9 Fig), which suggests that the dynamically changing contribution of CD8 effector cell killing could change the half-life of the second phase VL decay. By simulating VL dynamics based on estimated parameter with different CD8 effector cell killing rates, *m*, we found that strong CD8 T cell responses can reduce the half-life of second phase decay, while weak CD8 responses can prolong it (S14 Fig).

As previous models that did not include an effector cell response attributed the second phase VL decay after start of ART to the decay of long-lived infected cells [42], we built a model (see the *CTL-VC long-lived infected cell model* in SI for more details) to explicitly incorporate the long-lived infected cell population to fit the VL data. The *CTL-VC long-lived infected cell model* yielded slightly improved fits to the data over the original CTL-VC model (S15 Fig), but included four extra parameters whose values were approximated by scanning over a set of values. Predicted contributions to the VL dynamics based on estimated parameters from the *CTL-VC long-lived infected cell model* suggested the second phase of VL decay, which continues over a long period, mostly reflected the loss of cells in the latent reservoir (S16 Fig), while the loss of long-lived infected cells partly contributed to the VL during the transition from the first to the second phase. This is different than in HIV infection, where the loss of long-lived infected cells can explain second phase decay.

We computed the decay slope and half-life for the SIV latent reservoir based on the average frequencies of circulating resting CD4 T cells harboring replication-competent virus, which were measured as infectious cells per million cells (IUPM) at 4 different time points in 5 SIV-infected HAART suppressed monkeys as described on page 9251 and in Fig 5 in Dinoso et al. [30]. In humans on long-term suppressive ART, the latent reservoir decays with a half-life of 44 months [43, 44], which is too slow to explain the second phase decay. However, in SIV-infected macaques treated with short-term ART as in the Cartwright et al. study the latent reservoir seems to decay much faster, with an average half-life of about 38 days in the Dinoso et al. study [30], which is sufficiently fast to contribute substantially to second phase decay. However, we need to be cautious about this conclusion as the Dinoso et al. study used pigtail macaques, while the data analyzed here was obtained from rhesus macaques using a completely different ART regime than in the Dinoso et al. study. Simulation results from the *CTL-VC long-lived infected cell model* also show that the pre-CD8 depletion size of the latent reservoir, rather than the size of long-lived cell population, is responsible for the post-depletion peal viral load. Results from the *CTL-VC long-lived infected cell model* are consistent with the original CTL-VC model, and do not change the conclusions based on the CTL-VC model.

The Cartwright et al. study also included a set of data regarding virus sequencing at the time of peak viremia (“early virus”), immediately before ART initiation (“late virus”), and at the time of viremia rebound after CD8 depletion (“rebound virus”). In all study animals it was observed that the virus emerging after CD8 depletion is closely related to the virus circulating at the time of peak viremia and did not include the immune-escape mutations that accumulated in the SIV-Env gene during the interval between peak viremia (~day 10 post infection) and initiation of ART (day 56 post infection). These results of this longitudinal sequence analysis are not included in the current model of the mechanisms underlying virus control by CD8^+^ T cells under ART. However, it should be noted that the most parsimonious explanation for this pattern of sequence data is that the rebound of SIV production occurring after CD8^+^ lymphocyte depletion is due to latency reversal in cells that harbor “early virus” (as opposed to “late virus” that continued to replicate under ART). Further modeling studies in which the sequence data are taken into consideration will help assess the impact of latency reversal as a mechanism responsible for increased virus production after CD8 depletion.

Two experimental studies, in which CD8 T cells were depleted before ART, showed the VL decline slopes after initiation of ART were not changed by CD8 depletion when compared with undepleted controls [5, 6]. Based on these experiments, it was argued that the contribution of CD8 killing to suppressing viral replication in productively infected cells might be negligible. However, Klatt et al. [5] and Wong et al. [6] suggested that CD8 killing might still be occurring during the eclipse phase and Gadhamsetty et al. [8] suggested that the slope of VL decline is mainly determined by the rate of transit through the eclipse phase, rather than the rate of CD8 cytolytic killing. The CTL-VC model suggested a different mechanism. Specifically, due to the small contribution of CD8 cytolytic killing to cell death before ART, our model predicted that depletion of CD8 T cells before ART does not have a significant impact on the VL decay rate. However, although there were no significant changes in the decline slope, the VL was predicted to increase before ART in RMs with CD8 depleted one week before ART (Fig 6), consistent with the mean dynamics in Klatt et al. [5]. With explicit modeling of the CD8 effector and exhausted cell populations, our model provides a novel unified mechanism to explain the contribution of CD8 T cells in viral control. Overall, our modeling suggests that CD8 T cell cytolytic killing plays an important role in control of viral infection and that the magnitude of cytolytic killing is inversely dependent on the VL. Our models suggest a more important role of CD8 T cell cytolytic killing during ART and when VL is low. Therefore, future CD8 depletion experiments during the second phase VL decay might be useful to test these hypotheses, and also be useful to further study the role of CD8 T cells in viral control.

## Supporting information

S1 Text The *CTL-VC long-lived infected cell model*: CTL-VC model with a long-lived infected cell population. (DOCX)

S2 Text The *eclipse*-CTL-VC model: CTL-VC model with explicit modeling of the eclipse phase. (DOCX)

S1 Table Viral load data from the experiments in Cartwright et al. (10). DPI: days post infection. VL: viral load in unit SIV mRNA copies *mL*^−1^. Yellow cells are the VL measurements under ART with the first one the time of ART start. Orange cells indicate the time the CD8 depleting antibody was given.
(DOCX)

S2 Table CD8 count data from the experiments in Cartwright et al. (10). DPI: days post infection. CD8: CD8+ T cell count in unit of cells *μL*^−1^. Orange colored cells are measurements after CD8 depletion.
(DOCX)

S3 Table Estimated parameter values for the decay of the CD8 depleting antibody. (DOCX)

S4 Table BIC values of CTL-VC model fits with different *δ* values while fixing *β* = 3.0 × 10^−8^ *mL d*^−1^. The values of *δ* = 0.40 *d*^−1^ gives the smallest total BIC = 703. Highlights show the smallest BIC for each animal. Highlighted BIC with yellow color indicates it differs from the BIC obtained with *δ* = 0.40 *d*^−1^ by less than 2, which is not considered significant, while orange color indicates a difference larger than 2.
(DOCX)

S5 Table BIC values of CTL-VC model fits with different *β* values while fixing *δ* = 0.40 *d*^−1^. The value of *β* = 3.0 × 10^−8^ *mL d*^−1^ gives the smallest total BIC=703. Highlights show the smallest BIC for each animal. Highlighted BIC with yellow color indicates it differs from the BIC obtained with *β* = 3.0 × 10^−8^ *mL d*^−1^ by less than 2, which is not considered significant, while orange color indicates a difference larger than 2.
(DOCX)

S6 Table Estimate drug efficacy *ϵ* when fixing *α*_*L*_ at different values in CTL-VC model. (DOCX)

S7 Table Estimated parameter values for the CTL-VC model without cytolytic effects. (DOCX)

S8 Table Estimated parameter values for the CTL-VC model without non-cytolytic effects. (DOCX)

S9 Table Estimated parameter values for the *CTL-VC long-lived infected cell model* with *β*_*M*_ = 6 × 10^−9^ *mL d*^−1^, *δ*_*M*_ = 0.20 *d*^−1^ and *m*_*M*_ = 3 × 10^−5^ *mL cell*^−1^ *d*^−1^. (DOCX)

S10 Table Estimated parameter values for the *eclipse*-CTL-VC model. (DOCX)

S1 Fig The total CD8+ T cell count data (blue) and the viral load data (black) from Cartwright et al. (10). (TIF)

S2 Fig VL data from the CD8 depletion experiment (10). Black dots are the longitudinal VL data with connecting straight lines for eye guiding. The first vertical dashed line indicates the start time of ART, and the second vertical dashed line indicates the time of anti-CD8 antibody administration. The horizontal dashed line indicates the VL detection limit.
(TIF)

S3 Fig Total CD8 data from the CD8 depletion experiment (10). Blue dots are the longitudinal total CD8 count with connecting straight lines for eye guiding. The first vertical dashed line indicates the start time of ART, and the second vertical dashed line indicates the time of anti-CD8 antibody administration.
(TIF)

S4 Fig Fitting the logistic model to total CD8 count data to estimate CD8 depletion antibody decay parameters. (TIF)

S5 Fig Viral load dynamics predicted by the CTL-VC model based on estimated parameters (Table 2) with no CD8 depletion. The first vertical dashed line is the start time of ART, and the second vertical dashed line is the start time of CD8 depletion in the experiment.
(TIF)

S6 Fig Viral load dynamics predicted by the CTL-VC model based on estimated parameters (Table 2) with no ART. The first vertical dashed line is the start time of ART in the experiment, and the second vertical dashed line is the start time of CD8 depletion.
(TIF)

S7 Fig Dynamics of the total CD4^+^ T cell count. Black circles are CD4^+^ T cell count data from Cartwright et al. (10). Blue lines are the dynamics of total CD4^+^ T cell population calculated as the sum of target cell (T), latently infected cell (L) and the productively infected cell (I) from the CTL-VC model prediction.
(TIF)

S8 Fig Predicted dynamics of the SIV-specific CD8^+^ lymphocyte counts from the CTL-VC model. It consists of the sum of the effector cell population (E) and the exhausted cell population (X).
(TIF)

S9 Fig Predicted dynamics of the cytolytic killing rate *mE* (blue) with simulated viral dynamics (red) based on the estimated parameters from the CTL-VC model. (TIF)

S10 Fig Effective viral production rate *p*/(1 + *ηE*) (red lines) modulated by CD8 non-cytolytic effect from the CTL-VC model. Red lines: effective viral production rate. Black lines: the maximum viral production rate *p* estimated from data fitting.
(TIF)

S11 Fig Effective viral production rate *p*/(1 + *ηE*) modulated by CD8 non-cytolytic effect in the CTL-VC model with no CD8 cytolytic killing (*m* = 0). Red lines: effective viral production rate. Black lines: the maximum viral production rate *p* estimated from data fitting.
(TIF)

S12 Fig Slopes and half-lives of the second phase viral decay in intermediate and slow controllers. Two blue vertical lines indicate the time period for computing the second phase. Red line indicates the slope of second phase decay for each animal computed from VL data between the two blue vertical lines.
(TIF)

S13 Fig Contributions to the total viral load (black lines) by productively infected cells (red lines) and latently infected cells (green lines) in CTL-VC model. Slopes and half-lives are computed from the total viral load dynamics.
(TIF)

S14 Fig Predicted second phase viral decay at different strengths of CD8 effector cell response by changing the value of effector cell killing rate *m* in CTL-VC model. Black lines are the simulated viral load dynamics with the original value of *m* = 10^−4^ *mL cell*^−1^ *d*^−1^, while blue lines are simulations with a weaker CD8 effector cell response *m* = 5 × 10^−5^ *mL cell*^−1^ *d*^−1^ and red lines with a stronger CD8 response *m* = 2 × 10^−4^ *mL cell*^−1^ *d*^−1^.
(TIF)

S15 Fig Fitting the *CTL-VC long-lived infected cell model* to VL data. Red lines are the best model fits, and black dots are VL data points.
(TIF)

S16 Fig Predicted VL dynamics contributed by long-lived cells (blue lines), latently infected cells (green lines), and productively infected cells (red lines) according to the *CTL-VC long-lived infected cell model*. Black lines are the overall viral dynamics.
(TIF)

S17 Fig In the *CTL-VC long-lived infected cell model*, the correlation between (A) pre-depletion latent reservoir size and the post-depletion peak VL remains strong, while (B) the pre-depletion long-lived infected cell population also shows a correlation with the post-depletion peak VL.
(TIF)

S18 Fig Predicted CD8 cytolytic killing rate *mE* of the *CTL-VC long-lived infected cell model*. Red lines are the predicted effector cell cytolytic killing rate *mE*. The black horizontal lines are the fixed cytopathic death rate of infected cells, *δ* = 0.40 *d*^−1^.
(TIF)

S19 Fig Fitting the *eclipse*-CTL-VC model to the VL data. Red lines are model fits, and black dots are VL data points.
(TIF)
