# Supplementary material for: CD8+ lymphocyte control of SIV infection during antiretroviral therapy
Source: PLoS Pathog. 2018 Oct 11;14(10):e1007350. doi: 10.1371/journal.ppat.1007350 (PMC6199003; doi:10.1371/journal.ppat.1007350)
Supplement: S1 Text — (DOCX) [file ppat.1007350.s001.docx]

**S1 Text. The *CTL-VC long-lived infected cell model*: CTL-VC model with a long-lived infected cell population**

One interesting feature of CTL-VC model is that it captures the kinetics of the second phase of viral decay without introducing a population of long-lived infected cells. To study the possible contribution of long-lived cells to the second phase VL decay, we developed a new model, namely the *CTL-VC long-lived infected cell model* by explicitly incorporating the dynamics of infected long-lived cells ($M^{*}$) and their contributions to viral production into the original CTL-VC model: $\frac{dM^{*}}{dt}=\left( 1-\epsilon\right)\beta_{M}VM_{0}-\delta_{M}M^{*}-m_{M}EM^{*}$*,* $\frac{dV}{dt}=\frac{pI+p_{M}M^{*}}{1+\eta E}-cV$*,* where $\beta_{M}$ is the mass-action infectivity parameter for long-lived cells, $M_{0}$ is the density of uninfected long-lived cells, $\delta_{M}$ is the cytopathic death rate of infected long-lived cells, $m_{M}$ is the CD8 cytolytic killing rate for infected long-lived cells, and $p_{M}$ is the viral production rate from infected long-lived cells. According to Reilly et al. (1), we assume the viral production rate $p_{M}$ from long-lived cells is fivefold smaller than the production rate $p$ from actively infected cells: $p_{M}=\frac{p}{5}$. We assume that the infection rate $\beta_{M}$ is smaller for long-lived cells than for CD4 T cells and varied the value of $\beta_{M}$ among $3.0\times{10}^{-9} mL d^{-1}$, $6.0\times{10}^{-9} mL d^{-1}$ and $9.0\times{10}^{-9} mL d^{-1}$. We also varied the value of $\delta_{M}$ in $0<\delta_{M}<\delta=0.35 d^{-1}$, the value of $m_{M}$ in $0<m_{M}<m$ and the value of $M_{0}$ among${10}^{4}, {10}^{5}, \mathrm{and} {10}^{6} cells mL^{-1}$. We fit the *CTL-VC long-lived infected cell model* to the VL data and estimated the same 5 parameters as in the original CTL-VC model. Based on the total BIC in all 13 RMs, the parameter values $\beta_{M}=0.2\beta=6.0\times{10}^{-9} mL d^{-1}$, $\delta_{M}=0.5\delta=0.20 d^{-1}$, $m_{M}=0.3 m=3\times{10}^{-5} mL cell^{-1} d^{-1}$ and $M_{0}={10}^{5} cells mL^{-1}$ gave the best overall quality of fits (total BIC=689). Estimated parameters are listed in SI Table 9 and the model fits are shown in SI Figure 13. The quality of fits with the *CTL-VC long-lived infected cell model* is slightly improved over that of the original CTL-VC model (total BIC=703). Based on estimated parameters, the simulated dynamics of infected long-lived cells shows a mean per capita death rate $0.22 d^{-1}$ during the first 30 days after the start of ART, corresponding to a $t_{\frac{1}{2}}\approx3.2$days.

We compared the contributions of the three infected cell populations, *i.e.* long-lived infected cells, latently infected cells, and productively infected cells to the overall VL dynamics. To do so, we separately modeled the dynamics of virus produced from three different cell populations by expanding the *CTL-VC long-lived infected cell model* equations as following.

$$\frac{dT}{dt}=\lambda-d_{T}T-\left( 1-\epsilon\right)\beta(V_{0}+V_{1}+V_{M})T$$

$$\frac{dL}{dt}=\alpha_{L}\left( 1-\epsilon\right)\beta(V_{0}+V_{1}+V_{M})T+\left( \rho-a-d_{L} \right)L$$

$$\frac{dI_{0}}{dt}=\left( 1-\alpha_{L} \right)\left( 1-\epsilon\right)\beta(V_{0}+V_{1}+V_{M})T-\delta I_{0}-mEI_{0}$$

$$\frac{dI_{1}}{dt}=aL-\delta I_{1}-mEI_{1}$$

$$\frac{dM^{*}}{dt}=\left( 1-\epsilon\right)\beta_{M}(V_{0}+V_{1}+V_{M})M_{0}-\delta_{M}M^{*}-m_{M}EM^{*}$$

$$\frac{dV_{0}}{dt}=\frac{pI_{0}}{1+\eta E}-cV_{0}$$

$$\frac{dV_{1}}{dt}=\frac{pI_{1}}{1+\eta E}-cV_{1}$$

$$\frac{dV_{M}}{dt}=\frac{p_{M}M^{*}}{1+\eta E}-cV_{M}$$

$$\frac{dE}{dt}=\lambda_{E}+b_{E}\frac{I_{0}+I_{1}+M^{*}}{K_{B}+I_{0}+I_{1}+M^{*}} E-d_{E}\frac{I_{0}+I_{1}+M^{*}}{K_{D}+I_{0}+I_{1}+M^{*}} E-\mu E-k_{d}\frac{Ab\left( t \right)}{EC_{50}+Ab\left( t \right)}E$$

$$\frac{dX}{dt}=d_{E}\frac{I_{0}+I_{1}+M^{*}}{K_{D}+I_{0}+I_{1}+M^{*}} E-0.5\mu X-k_{d}\frac{Ab\left( t \right)}{EC_{50}+Ab\left( t \right)}X$$

$V_{0}$ is the VL contributed by productively infected cells, $V_{1}$ is contributed by latently infected cells, and $V_{M}$ is contributed by the long-lived cells. Based on the estimated parameters from the *CTL-VC long-lived infected cell model*, the simulated trajectories of $V_{0}$, $V_{1}$, $V_{M}$ and the total viral load ($V_{0}+V_{1}+V_{M}$) are shown in SI Figure 14. Based on our simulations, productively infected cells dominantly contributed to the VL during the first phase VL decay for about 2 weeks after initiation of ART with a mean slope of $0.35 d^{-1}$ and mean half-life about 2 days (SI Figure 14 red lines). During the second VL decay phase, the latent reservoir is the major contributor with a mean decay slope of $0.031 d^{-1}$ and a mean half-life about 23 days (SI Figure 14 green lines). The long-lived infected cells contributed to the VL decay between the first and the second phase with a mean slope of $0.23 d^{-1}$ and mean half-life about $3.0$ days (SI Figure 14 blue lines).

To examine the impact of incorporating the long-lived cell population on the conclusions, we computed the correlation between pre-depletion latent reservoir size and post-depletion peak VL based on estimated parameters from the *CTL-VC long-lived infected cell model*. The correlation between the pre-depletion latent reservoir size and post-depletion peak VL remains strong and statistically significant ($R=0.74$ and $p=0.004$) (SI Figure 15A). While the pre-depletion long-lived cell population size show a weaker correlation with the post-depletion peak VL ($R=0.64$ and $p=0.018$) (SI Figure 15B), the magnitude of VL contribution from long-lived cell population is 2-3 logs smaller than latency activation. Furthermore, the predicted CD8 cytolytic killing rates for both productively $mE$ and long-lived $m_{M}E$ infected cells of the *long-lived*-CTL VC model appears to have the same patterns as those of the original CTL-VC model while having larger magnitude (SI Figure 16), *i.e.* $mE$ increases after the start of ART and CD8 cytolytic killing plays an important role during ART. Overall, results from the *CTL-VC long-lived infected cell model* are compatible with the conclusions based on the original CTL-VC model.

References

1. Reilly C, Wietgrefe S, Sedgewick G, Haase A. Determination of simian immunodeficiency virus production by infected activated and resting cells. AIDS. 2007;21(2):163-8.
