# Supplementary material for: CD8+ lymphocyte control of SIV infection during antiretroviral therapy
Source: PLoS Pathog. 2018 Oct 11;14(10):e1007350. doi: 10.1371/journal.ppat.1007350 (PMC6199003; doi:10.1371/journal.ppat.1007350)
Supplement: S2 Text — (DOCX) [file ppat.1007350.s002.docx]

**S2 Text. The *eclipse*-CTL-VC model: CTL-VC model with explicit modeling of the eclipse phase**

To study the effect of the eclipse phase, we developed a new model, namely the *eclipse*-CTL-VC model by explicitly incorporating the eclipse phase of HIV infection into the original CTL-VC model. Specifically, we include an additional compartment $C$ for cells in eclipse phase:

$\frac{dC}{dt}=\left( 1-\epsilon\right) \beta VT -\frac{1}{t_{C}}C-d_{C}C-m_{C}EC$

$\frac{dL}{dt}=\alpha_{L}\frac{1}{t_{C}}C+\left( \rho- a - d_{L} \right)L$

$\frac{dI}{dt}=\left( 1-\alpha_{L} \right)\frac{1}{t_{C}}C-\delta I+aL-mEI$,

where $t_{C}$ is the duration of the eclipse phase, $d_{C}$ is the natural per capita death rate and $m_{C}$ is the CD8 T cell cytolytic killing rate for infected cells during eclipse phase. We fit the *eclipse*-CTL-VC model to the VL data by estimating the length of the eclipse phase $0.1<t_{C}<5 days$ and the same 5 parameters as in the CTL-VC model. We scan through different values of $d_{C}$, $m_{C}$ and $\delta$ to find the best quality of fits. We choose the value $\delta=2.20 d^{-1}$, $d_{C}=0 d^{-1}$ and $m_{C}=0 mL cell^{-1} d^{-1}$, which gives the best overall fitting quality (total BIC=727). Estimated parameters for the *eclipse*-CTL-VC model are listed in SI Table 10 and the fits for each RM are shown in SI Figure 17.

Results show that the *eclipse*-CTL-VC model can also fit the data well (SI Figure 17) with a total log-likelihood (total $LL=-229$) close to that of the CTL-VC model (total $LL=-217$), although the best fit (total BIC=727) is slightly worse than that of the original CTL-VC model (total BIC=703). Additionally, we found that estimated viral production rates $p$ of the *eclipse*-CTL-VC model (mean $p=19,232 d^{-1}$) (SI Table 10) are significantly larger than those of CTL-VC model (mean $p=4,046 d^{-1}$), and the cytopathic death rate $\delta=2.20 d^{-1}$ that gives the best fit using the *eclipse*-CTL-VC model is also larger than the best-fit value of $\delta=0.40 d^{-1}$ from the CTL-VC model. A higher cytopathic death rate using a model with an eclipse phase than that from model without an eclipse phase was also found by Gadhamsetty *et al* (1). This difference can be explained because $\delta$ in the CTL-VC model covers the lifespan of both eclipse phase and productive phase of infected cells, while $\delta$ in the *eclipse*-CTL-VC model only covers the productive phase. The value of $\delta=2.20 d^{-1}$ in the *eclipse*-CTL-VC model corresponds to a mean half-life of $-\frac{\log\left( 0.5 \right)}{2.20}\approx0.32 d$ for the productive phase. With an additional 1.93 days of average lifespan ($t_{E}$) in eclipse phase (SI Table 10), the total mean lifespan of an infected cell is about 2.25 days, which corresponds to a lumped cytopathic death rate $-\frac{\log0.5}{2.25}\approx0.31 d^{-1}$, close to the value $\delta=0.40 d^{-1}$ estimated with the CTL-VC model. Similarly, in the eclipse phase model viral production occurs over a shorter interval and hence the viral production rate, $p$, is higher. In fact, the predicted viral burst size, $p/\delta$, is the same for both models ~$9.0\times{10}^{3}$ virions $d^{-1}$. The results from the *eclipse*-CTL-VC model are consistent with the results from the CTL-VC model, in that both models showed a VL increase and similar first phase VL decline slope after ART initiation when CD8 T cells are depleted about one week before ART (Figure 6A and B), and both model fits exhibited low CD8 cytolytic killing rates before ART in most macaques (Figure 6C and D).

References

1. Gadhamsetty S, Beltman JB, de Boer RJ. What do mathematical models tell us about killing rates during HIV-1 infection? Immunol Lett. 2015;168(1):1-6.
