# Supplementary material for: CD8+ lymphocyte control of SIV infection during antiretroviral therapy
Source: PLoS Pathog. 2018 Oct 11;14(10):e1007350. doi: 10.1371/journal.ppat.1007350 (PMC6199003; doi:10.1371/journal.ppat.1007350)
Supplement: S1 Table — DPI: days post infection. VL: viral load in unit SIV mRNA copies mL−1. Yellow cells are the VL measurements under ART with the first one the time of ART start. Orange cells indicate the time the CD8 depleting antibody was given. (DOCX) [file ppat.1007350.s003.docx]

**SI Table 1. Viral load data from the experiments in Cartwright et al. (1). DPI: days post infection. VL: viral load in unit SIV mRNA copies** $\boldsymbol{m}\boldsymbol{L}^{\boldsymbol{-1}}$**. Yellow cells are the VL measurements under ART with the first one the time of ART start. Orange cells indicate the time the CD8 depleting antibody was given.**

| **RGb13** | | **RLb13** | | **RVy10** | | **RKq11** | | **ROw8** | |
| --- | --- | --- | --- | --- | --- | --- | --- | --- | --- |
| **DPI** | **VL** | **DPI** | **VL** | **DPI** | **VL** | **DPI** | **VL** | **DPI** | **VL** |
| 7 | 534000 | 7 | 3950000 | 7 | 3000000 | 7 | 2880000 | 7 | 2340000 |
| 10 | 9020000 | 10 | 27200000 | 10 | 59600000 | 10 | 17700000 | 10 | 16300000 |
| 14 | 1470000 | 14 | 4740000 | 14 | 13800000 | 14 | 3040000 | 14 | 968000 |
| 21 | 185000 | 21 | 788000 | 21 | 2120000 | 21 | 2920000 | 21 | 1260000 |
| 28 | 6000 | 28 | 80000 | 28 | 1120000 | 28 | 784000 | 28 | 388000 |
| 42 | 15200 | 42 | 248000 | 42 | 165000 | 42 | 302000 | 42 | 61900 |
| 56 | 413 | 56 | 233000 | 56 | 2940000 | 56 | 599000 | 56 | 124000 |
| 72 | 30 | 72 | 94.8 | 72 | 4100 | 72 | 1200 | 72 | 30 |
| 79 | 30 | 79 | 30 | 79 | 1120 | 79 | 344 | 79 | 30 |
| 93 | 30 | 93 | 30 | 93 | 30 | 93 | 176 | 93 | 30 |
| 107 | 30 | 107 | 30 | 107 | 239 | 107 | 192 | 107 | 30 |
| 112 | 30 | 112 | 30 | 114 | 30 | 114 | 176 | 112 | 30 |
| 119 | 30 | 119 | 30 | 121 | 206 | 121 | 30 | 119 | 30 |
| 120 | 30 | 120 | 30 | 128 | 30 | 128 | 206 | 120 | 85 |
| 121 | 30 | 121 | 152 | 135 | 103 | 135 | 30 | 121 | 30 |
| 126 | 30 | 126 | 271 | 142 | 30 | 156 | 30 | 126 | 519 |
| 133 | 30 | 133 | 30 | 149 | 30 | 163 | 30 | 133 | 268 |
| 140 | 91 | 140 | 334 | 156 | 30 | 170 | 30 | 140 | 454 |
| 154 | 30 | 154 | 30 | 170 | 30 | 184 | 30 | 154 | 941 |
| 161 | 30 | 161 | 30 | 184 | 30 | 198 | 30 | 161 | 386 |
| 170 | 30 | 170 | 30 | 191 | 30 | 205 | 30 | 170 | 212 |
| 180 | 30 | 180 | 30 | 192 | 30 | 206 | 30 |  |  |
|  |  |  |  | 193 | 85.8 | 207 | 99 |  |  |
|  |  |  |  | 198 | 83.7 | 212 | 79.7 |  |  |
|  |  |  |  | 205 | 178 | 219 | 210 |  |  |
|  |  |  |  | 212 | 300 | 226 | 60 |  |  |
|  |  |  |  | 226 | 30 | 240 | 444 |  |  |
|  |  |  |  |  |  | 247 | 546 |  |  |
|  |  |  |  |  |  | 254 | 240 |  |  |
|  |  |  |  |  |  | 261 | 30 |  |  |
|  |  |  |  |  |  |  |  |  |  |

*SI Table 1. Continued.*

| **RBv13** | | **RWj14** | | **RYF14** | | **RAz12** | | **RSj14** | |
| --- | --- | --- | --- | --- | --- | --- | --- | --- | --- |
| **DPI** | **VL** | **DPI** | **VL** | **DPI** | **VL** | **DPI** | **VL** | **DPI** | **VL** |
| 7 | 5700000 | 7 | 2000000 | 7 | 10000000 | 7 | 2690000 | 7 | 12600000 |
| 10 | 48200000 | 10 | 12500000 | 10 | 66600000 | 10 | 35200000 | 10 | 42000000 |
| 14 | 7120000 | 14 | 6430000 | 14 | 6290000 | 14 | 12400000 | 14 | 4210000 |
| 21 | 3480000 | 21 | 1010000 | 21 | 2060000 | 21 | 3920000 | 21 | 2090000 |
| 28 | 2880000 | 28 | 428000 | 28 | 1100000 | 28 | 2000000 | 28 | 500000 |
| 42 | 1700000 | 42 | 504000 | 42 | 2120000 | 42 | 3020000 | 42 | 1040000 |
| 56 | 1580000 | 56 | 496000 | 57 | 3400000 | 56 | 2270000 | 56 | 921000 |
| 72 | 2300 | 72 | 2150 | 72 | 7550 | 72 | 11300 | 72 | 5260 |
| 79 | 426 | 79 | 984 | 79 | 4200 | 79 | 6280 | 79 | 2720 |
| 93 | 479 | 93 | 584 | 93 | 562 | 93 | 5370 | 93 | 1890 |
| 107 | 215 | 107 | 334 | 107 | 1600 | 107 | 1340 | 107 | 397 |
| 114 | 1120 | 114 | 157 | 114 | 403 | 114 | 705 | 114 | 296 |
| 121 | 303 | 128 | 101 | 121 | 372 | 121 | 677 | 121 | 508 |
| 128 | 240 | 135 | 481 | 128 | 156 | 128 | 622 | 128 | 440 |
| 135 | 218 | 142 | 235 | 135 | 156 | 135 | 460 | 135 | 213 |
| 149 | 110 | 149 | 379 | 142 | 329 | 142 | 494 | 142 | 327 |
| 156 | 30 | 156 | 93.4 | 149 | 75 | 149 | 130 | 149 | 117 |
| 163 | 156 | 163 | 30 | 156 | 96.4 | 156 | 137 | 156 | 239 |
| 170 | 30 | 170 | 30 | 163 | 30 | 163 | 267 | 163 | 651 |
| 177 | 30 | 184 | 30 | 170 | 164 | 170 | 40200 | 170 | 151 |
| 184 | 30 | 191 | 30 | 177 | 30 | 177 | 113 | 177 | 207 |
| 198 | 30 | 192 | 135 | 184 | 30 | 184 | 213 | 184 | 290 |
| 205 | 30 | 193 | 303 | 198 | 134 | 191 | 145 | 191 | 344 |
| 206 | 66 | 198 | 766 | 205 | 111 | 198 | 277 | 198 | 271 |
| 207 | 146 | 205 | 1040 | 206 | 161 | 205 | 30 | 205 | 101 |
| 212 | 174 | 212 | 446 | 207 | 156 | 212 | 152 | 212 | 196 |
| 219 | 30 | 226 | 30 | 212 | 192 | 219 | 124 | 219 | 30 |
| 226 | 203.7299 | 233 | 86 | 219 | 538 | 226 | 79 | 226 | 116 |
| 240 | 136 | 240 | 142 | 226 | 193 | 233 | 30 | 233 | 30 |
| 247 | 30 | 247 | 30 | 240 | 481 | 240 | 66.8 | 240 | 30 |
| 254 | 462 |  |  | 247 | 119 | 247 | 72.4 | 247 | 63.9 |
| 261 | 30 |  |  | 254 | 284 | 261 | 30 | 261 | 30 |
|  |  |  |  | 261 | 85 | 268 | 142 | 268 | 30 |
|  |  |  |  |  |  | 269 | 287 | 269 | 30 |
|  |  |  |  |  |  | 270 | 253 | 270 | 178 |
|  |  |  |  |  |  | 275 | 531 | 275 | 261 |
|  |  |  |  |  |  | 282 | 572 | 282 | 667 |
|  |  |  |  |  |  | 289 | 741 | 289 | 424 |
|  |  |  |  |  |  | 303 | 281 | 303 | 1380 |
|  |  |  |  |  |  | 310 | 486 | 310 | 108 |
|  |  |  |  |  |  | 317 | 30 | 317 | 91 |
|  |  |  |  |  |  | 324 | 30 | 324 | 30 |

*SI Table 1. Continued.*

| **RDh10** | | **RLc10** | | **ROn13** | |
| --- | --- | --- | --- | --- | --- |
| **DPI** | **VL** | **DPI** | **VL** | **DPI** | **VL** |
| 7 | 1000000 | 7 | 2060000 | 7 | 800000 |
| 10 | 13800000 | 10 | 22200000 | 10 | 31600000 |
| 14 | 5830000 | 14 | 18800000 | 14 | 48600000 |
| 21 | 908000 | 21 | 5780000 | 21 | 4110000 |
| 28 | 629000 | 28 | 2040000 | 28 | 7360000 |
| 42 | 1590000 | 42 | 2970000 | 42 | 14600000 |
| 57 | 3400000 | 56 | 1940000 | 57 | 112000000 |
| 72 | 25300 | 72 | 8220 | 72 | 386000 |
| 79 | 13400 | 79 | 2480 | 79 | 90800 |
| 93 | 324 | 93 | 1760 | 93 | 34300 |
| 107 | 478 | 107 | 1660 | 107 | 20400 |
| 114 | 248 | 114 | 1880 | 114 | 13300 |
| 121 | 163 | 121 | 848 | 121 | 19100 |
| 128 | 65 | 128 | 934 | 128 | 8500 |
| 135 | 62.9 | 135 | 736 | 135 | 9530 |
| 142 | 265 | 142 | 671 | 142 | 3390 |
| 149 | 561 | 149 | 510 | 149 | 2630 |
| 156 | 73.8 | 156 | 504 | 156 | 2030 |
| 163 | 75.2 | 163 | 253 | 163 | 4140 |
| 170 | 97.2 | 170 | 303 | 170 | 3590 |
| 177 | 181 | 177 | 369 | 177 | 3920 |
| 184 | 171 | 184 | 30 | 184 | 2680 |
| 191 | 242 | 191 | 214 | 191 | 1570 |
| 198 | 209 | 198 | 153 | 198 | 1640 |
| 205 | 30 | 205 | 282 | 205 | 928 |
| 212 | 155 | 212 | 30 | 212 | 857 |
| 219 | 182 | 219 | 76 | 219 | 663 |
| 226 | 30 | 226 | 122 | 226 | 1210 |
| 233 | 30 | 233 | 194 | 233 | 1080 |
| 247 | 30 | 240 | 108 | 240 | 717 |
| 254 | 81 | 254 | 30 | 247 | 986 |
| 255 | 286 | 268 | 106 | 254 | 700 |
| 256 | 386 | 275 | 88.6 | 268 | 529 |
| 261 | 240 | 276 | 1770 | 275 | 530 |
| 268 | 121 | 277 | 222 | 282 | 326 |
| 282 | 438 | 282 | 913 | 283 | 437 |
| 289 | 284 | 289 | 2090 | 284 | 805 |
| 296 | 30 | 296 | 1660 | 289 | 1090 |
| 303 | 30 | 310 | 2660 | 296 | 12500 |
| 310 | 30 | 317 | 1640 | 303 | 107000 |
|  |  | 324 | 603 | 317 | 385000 |
|  |  |  |  | 324 | 101000 |
|  |  |  |  | 331 | 98900 |
|  |  |  |  | 338 | 29000 |

1. Cartwright EK, Spicer L, Smith SA, Lee D, Fast R, Paganini S, et al. CD8(+) lymphocytes are required for maintaining viral suppression in SIV-infected macaques treated with short-term antiretroviral therapy. Immunity. 2016;45(3):656-68.
