# Supplementary material for: CD8+ lymphocyte control of SIV infection during antiretroviral therapy
Source: PLoS Pathog. 2018 Oct 11;14(10):e1007350. doi: 10.1371/journal.ppat.1007350 (PMC6199003; doi:10.1371/journal.ppat.1007350)
Supplement: S2 Table — DPI: days post infection. CD8: CD8+ T cell count in unit of cells μL−1. Orange colored cells are measurements after CD8 depletion. (DOCX) [file ppat.1007350.s004.docx]

**SI Table 2. CD8 count data from the experiments in Cartwright et al. (1). DPI: days post infection. CD8: CD8+ T cell count in unit of cells** $\boldsymbol{\mu}\boldsymbol{L}^{\boldsymbol{-1}}$**. Orange colored cells are measurements after CD8 depletion.**

| **RGb13** | | **RLb13** | | **RVy10** | | **RKq11** | | **ROw8** | | **RBv13** | | **RWj14** | |
| --- | --- | --- | --- | --- | --- | --- | --- | --- | --- | --- | --- | --- | --- |
| **DPI** | **CD8** | **DPI** | **CD8** | **DPI** | **CD8** | **DPI** | **CD8** | **DPI** | **CD8** | **DPI** | **CD8** | **DPI** | **CD8** |
| 14 | 436 | 14 | 486 | 14 | 1400 | 14 | 702 | 14 | 1690 | 14 | 2070 | 14 | 588 |
| 28 | 872 | 28 | 804 | 28 | 628 | 28 | 805 | 28 | 647 | 28 | 3240 | 28 | 276 |
| 56 | 519 | 56 | 1360 | 56 | 397.5 | 56 | 600 | 56 | 905 | 56 | 1440 | 56 | 507 |
| 112 | 552 | 112 | 820 | 184 | 682 | 198 | 1480 | 112 | 1520 | 198 | 1810 | 184 | 895 |
| 120 | 30.5 | 120 | 85.2 | 192 | 51.6 | 206 | 34.4 | 120 | 0.383 | 206 | 34.5 | 192 | 104 |
| 121 | 13.8 | 121 | 27.4 | 193 | 29.9 | 207 | 58.5 | 121 | 0.995 | 207 | 27.1 | 193 | 74.2 |
| 126 | 2.05 | 126 | 29.3 | 198 | 68.7 | 212 | 70.8 | 126 | 0.031 | 212 | 14 | 198 | 98.2 |
| 133 | 1.14 | 133 | 13.2 | 205 | 44.5 | 219 | 0.077 | 133 | 51.3 | 219 | 0.205 | 205 | 89.7 |
| 140 | 45.5 | 140 | 28 | 212 | 22.6 | 226 | 59.4 | 140 | 108 | 226 | 22.2 | 212 | 37.6 |
| 154 | 471 | 154 | 734 | 226 | 594 | 240 | 60.8 | 154 | 15.02 | 240 | 399 | 226 | 351 |
| 161 | 221 | 161 | 205 |  |  | 247 | 81.6 | 161 | 101 | 247 | 94 | 233 | 415 |
| 170 | 538 | 170 | 497 |  |  | 254 | 126 | 170 | 42.1 | 254 | 817 | 240 | 377 |
| 180 | 503 | 180 | 570 |  |  | 261 | 511 |  |  | 261 | 543 | 247 | 266 |
|  |  |  |  |  |  |  |  |  |  |  |  |  |  |
| **RYF14** | | **RAz12** | | **RSj14** | | **RDh10** | | **RLc10** | | **ROn13** | |  |  |
| **DPI** | **CD8** | **DPI** | **CD8** | **DPI** | **CD8** | **DPI** | **CD8** | **DPI** | **CD8** | **DPI** | **CD8** |  |  |
| 14 | 1180 | 14 | 1050 | 14 | 1580 | 14 | 989 | 14 | 469 | 14 | 641 |  |  |
| 28 | 671 | 28 | 544 | 28 | 1300 | 28 | 533 | 28 | 188 | 28 | 1300 |  |  |
| 57 | 701 | 56 | 552.7 | 56 | 740 | 57 | 701 | 56 | 305 | 57 | 1314 |  |  |
| 198 | 1070 | 261 | 1570 | 261 | 1350 | 247 | 833 | 268 | 245 | 275 | 1140 |  |  |
| 206 | 57.8 | 269 | 70.8 | 269 | 68.7 | 255 | 2.14 | 276 | 0.311 | 283 | 0.558 |  |  |
| 207 | 61.5 | 270 | 38.6 | 270 | 41.6 | 256 | 0.386 | 277 | 0.869 | 284 | 1.06 |  |  |
| 212 | 147 | 275 | 135 | 275 | 2.67 | 261 | 4.44 | 282 | 1.25 | 289 | 2.26 |  |  |
| 219 | 28.7 | 282 | 57.1 | 282 | 4.94 | 268 | 3.25 | 289 | 3.04 | 296 | 3.03 |  |  |
| 226 | 49.3 | 289 | 67.5 | 289 | 13.3 | 282 | 2.9 | 296 | 7.3 | 303 | 5.37 |  |  |
| 240 | 78.1 | 303 | 124 | 303 | 501 | 289 | 12.6 | 310 | 0.697 | 317 | 1.78 |  |  |
| 247 | 33.9 | 310 | 767 | 310 | 488 | 296 | 6.42 | 317 | 4.46 | 324 | 6.28 |  |  |
| 254 | 332 | 317 | 686 | 317 | 637 | 303 | 20.8 | 324 | 9.06 | 331 | 9.41 |  |  |
| 261 | 522 | 324 | 482 | 324 | 453 | 310 | 17.7 |  |  | 338 | 7.99 |  |  |

1. Cartwright EK, Spicer L, Smith SA, Lee D, Fast R, Paganini S, et al. CD8(+) lymphocytes are required for maintaining viral suppression in SIV-infected macaques treated with short-term antiretroviral therapy. Immunity. 2016;45(3):656-68.
