# Supplementary material for: CD8+ lymphocyte control of SIV infection during antiretroviral therapy
Source: PLoS Pathog. 2018 Oct 11;14(10):e1007350. doi: 10.1371/journal.ppat.1007350 (PMC6199003; doi:10.1371/journal.ppat.1007350)
Supplement: S3 Table — (DOCX) [file ppat.1007350.s005.docx]

**SI Table 3. Estimated parameter values for the decay of the CD8 depleting antibody.**

| RM | $\boldsymbol{k}_{\boldsymbol{d}}$ ($\boldsymbol{d}^{\boldsymbol{-1}}$) | $\boldsymbol{k}_{\boldsymbol{1}}$ ($\boldsymbol{d}^{\boldsymbol{-1}}$) | $\boldsymbol{k}_{\boldsymbol{2}}$ ($\boldsymbol{d}^{\boldsymbol{-1}}$) | -LL |
| --- | --- | --- | --- | --- |
| RGb13 | 7.58 | 2.71E-02 | 2.70E-02 | 0.50 |
| RLb13 | 6.08 | 2.25E-02 | 2.24E-02 | 0.56 |
| ROw8 | 14.67 | 1.30E-02 | 2.62E-01 | 12.12 |
| RVy10 | 6.42 | 1.64E-02 | 1.64E-02 | 1.17 |
| RKq11 | 6.51 | 1.07E-02 | 4.56E-01 | 6.77 |
| RBv13 | 9.23 | 1.49E-02 | 2.57E+00 | 5.21 |
| RWj14 | 5.42 | 7.07E-02 | 1.63E-02 | 0.44 |
| RYF14 | 6.03 | 5.18E-02 | 1.04E-02 | 0.85 |
| RAz12 | 5.84 | 4.83E-01 | 1.38E-02 | 0.81 |
| RSj14 | 7.88 | 1.47E-02 | 1.02E+00 | 1.29 |
| RDh10 | 10.95 | 2.59E-02 | 1.00E-02 | 2.51 |
| RLc10 | 12.28 | 1.06E-02 | 4.02E-02 | 2.36 |
| ROn13 | 11.49 | 7.79E-02 | 1.00E-02 | 2.42 |
