# Supplementary material for: CD8+ lymphocyte control of SIV infection during antiretroviral therapy
Source: PLoS Pathog. 2018 Oct 11;14(10):e1007350. doi: 10.1371/journal.ppat.1007350 (PMC6199003; doi:10.1371/journal.ppat.1007350)
Supplement: S4 Table — The values of δ = 0.40 d−1 gives the smallest total BIC = 703. Highlights show the smallest BIC for each animal. Highlighted BIC with yellow color indicates it differs from the BIC obtained with δ = 0.40 d−1 by less than 2, which is not considered significant, while orange color indicates a difference larger than 2. (DOCX) [file ppat.1007350.s006.docx]

**SI Table 4. BIC values of CTL-VC model fits with different** $\boldsymbol{\delta}$ **values while fixing** $\boldsymbol{\beta=3.0\times}\boldsymbol{10}^{\boldsymbol{-8}}\boldsymbol{mL}\boldsymbol{d}^{\boldsymbol{-1}}$**. The value of** $\boldsymbol{\delta=0.40}\boldsymbol{d}^{\boldsymbol{-1}}$ **gives the smallest total BIC=703. Highlights show the smallest BIC for each animal. Highlighted BIC with yellow color indicates it differs from the BIC obtained with** $\boldsymbol{\delta=0.40}\boldsymbol{d}^{\boldsymbol{-1}}$ **by less than 2, which is not considered significant, while orange color indicates a difference larger than 2.**

| $\boldsymbol{\delta}$ | **0.15** | **0.20** | **0.25** | **0.30** | **0.35** | **0.40** | **0.45** | **0.50** | **0.55** |
| --- | --- | --- | --- | --- | --- | --- | --- | --- | --- |
| **RGb13** | 12.35 | 10.73 | 9.88 | 9.70 | 9.90 | 10.20 | 10.54 | 10.87 | 11.18 |
| **RLb13** | 13.36 | 12.17 | 11.25 | 10.63 | 10.33 | 10.40 | 10.93 | 11.91 | 13.22 |
| **ROw8** | 12.25 | 10.80 | 9.94 | 9.55 | 9.59 | 10.15 | 11.26 | 11.91 | 13.34 |
| **RVy10** | 19.18 | 18.37 | 18.10 | 17.42 | 17.02 | 16.71 | 16.56 | 17.92 | 20.01 |
| **RKq11** | 21.74 | 19.50 | 17.90 | 17.96 | 17.22 | 16.56 | 16.31 | 17.95 | 18.70 |
| **RBv13** | 27.89 | 25.64 | 25.25 | 24.18 | 23.89 | 23.66 | 23.48 | 23.67 | 24.04 |
| **RWj14** | 25.80 | 21.63 | 18.87 | 17.52 | 16.98 | 17.02 | 17.63 | 18.80 | 19.01 |
| **RYF14** | 23.23 | 17.76 | 15.41 | 10.48 | 8.22 | 7.10 | 8.08 | 8.79 | 9.33 |
| **RAz12** | 40.38 | 37.75 | 35.35 | 31.97 | 29.54 | 28.27 | 27.72 | 27.56 | 27.68 |
| **RSj14** | 28.12 | 23.03 | 19.57 | 16.89 | 14.64 | 12.49 | 13.11 | 16.97 | 19.11 |
| **RDh10** | 34.33 | 29.91 | 27.66 | 26.42 | 25.48 | 25.80 | 27.10 | 28.63 | 30.07 |
| **RLc10** | 28.35 | 25.63 | 25.09 | 20.57 | 16.75 | 14.56 | 14.08 | 14.26 | 14.45 |
| **ROn13** | 23.69 | 20.60 | 20.20 | 21.25 | 30.97 | 23.88 | 33.87 | 34.99 | 35.85 |
| **Total** | 891.04 | 816.71 | 778.66 | 738.78 | 730.78 | 703.32 | 731.03 | 758.16 | 781.68 |
