# Supplementary material for: CD8+ lymphocyte control of SIV infection during antiretroviral therapy
Source: PLoS Pathog. 2018 Oct 11;14(10):e1007350. doi: 10.1371/journal.ppat.1007350 (PMC6199003; doi:10.1371/journal.ppat.1007350)
Supplement: S5 Table — The value of β = 3.0 × 10−8 mL d−1 gives the smallest total BIC=703. Highlights show the smallest BIC for each animal. Highlighted BIC with yellow color indicates it differs from the BIC obtained with β = 3.0 × 10−8 mL d−1 by less than 2, which is not considered significant, while orange color indicates a difference larger than 2. (DOCX) [file ppat.1007350.s007.docx]

**SI Table 5. BIC values of CTL-VC model fits with different** $\boldsymbol{\beta}$ **values while fixing** $\boldsymbol{\delta=0.40}\boldsymbol{d}^{\boldsymbol{-1}}$**. The value of** $\boldsymbol{\beta=3.0\times}\boldsymbol{10}^{\boldsymbol{-8}}\boldsymbol{mL}\boldsymbol{d}^{\boldsymbol{-1}}$ **gives the smallest total BIC=703. Highlights show the smallest BIC for each animal. Highlighted BIC with yellow color indicates it differs from the BIC obtained with**$\boldsymbol{\beta=3.0\times}\boldsymbol{10}^{\boldsymbol{-8}}\boldsymbol{mL}\boldsymbol{d}^{\boldsymbol{-1}}$ **by less than 2, which is not considered significant, while orange color indicates a difference larger than 2.**

| $\boldsymbol{\beta}$ | **1.50E-08** | **2.00E-08** | **2.50E-08** | **3.00E-08** | **3.50E-08** | **4.00E-08** | **4.50E-08** |
| --- | --- | --- | --- | --- | --- | --- | --- |
| **RGb13** | 10.49 | 10.38 | 10.27 | 10.20 | 10.18 | 10.22 | 10.18 |
| **RLb13** | 11.19 | 10.56 | 10.36 | 10.40 | 10.58 | 10.84 | 11.14 |
| **ROw8** | 12.20 | 10.73 | 10.31 | 10.15 | 10.14 | 10.23 | 10.38 |
| **RVy10** | 15.85 | 16.04 | 16.37 | 16.71 | 17.01 | 17.19 | 17.32 |
| **RKq11** | 17.04 | 16.63 | 16.52 | 16.56 | 16.69 | 16.85 | 17.03 |
| **RBv13** | 23.75 | 24.02 | 23.85 | 23.66 | 23.61 | 23.68 | 23.81 |
| **RWj14** | 17.73 | 17.27 | 17.07 | 17.02 | 17.05 | 17.13 | 17.24 |
| **RYF14** | 9.16 | 7.75 | 7.18 | 7.10 | 7.30 | 7.57 | 7.75 |
| **RAz12** | 30.67 | 29.49 | 28.75 | 28.27 | 27.96 | 27.78 | 27.71 |
| **RSj14** | 15.42 | 14.97 | 13.62 | 12.49 | 11.80 | 11.45 | 11.34 |
| **RDh10** | 27.61 | 26.56 | 26.02 | 25.80 | 25.78 | 25.90 | 26.10 |
| **RLc10** | 17.61 | 15.79 | 14.90 | 14.56 | 14.53 | 14.69 | 14.97 |
| **ROn13** | 26.36 | 21.49 | 22.69 | 23.88 | 33.68 | 34.71 | 35.62 |
| **Total** | 739.85 | 713.04 | 705.53 | 703.32 | 722.35 | 726.17 | 730.86 |
