# Supplementary material for: CD8+ lymphocyte control of SIV infection during antiretroviral therapy
Source: PLoS Pathog. 2018 Oct 11;14(10):e1007350. doi: 10.1371/journal.ppat.1007350 (PMC6199003; doi:10.1371/journal.ppat.1007350)
Supplement: S6 Table — (DOCX) [file ppat.1007350.s008.docx]

**SI Table 6. Estimate drug efficacy** $\boldsymbol{\epsilon}$ **when fixing** $\boldsymbol{\alpha}_{\boldsymbol{L}}$ **at different values in CTL-VC model.**

| **RM** | $\boldsymbol{\alpha}_{\boldsymbol{L}}$ | $\boldsymbol{\epsilon}$ | $\boldsymbol{p}$ ($\boldsymbol{virions cel}\boldsymbol{l}^{\boldsymbol{-1}}\boldsymbol{d}^{\boldsymbol{-1}}$) | $\boldsymbol{d}_{\boldsymbol{E}}$ ($\boldsymbol{cells m}\boldsymbol{L}^{\boldsymbol{-1}}\boldsymbol{d}^{\boldsymbol{-1}}$) | $\boldsymbol{K}_{\boldsymbol{B}}$ $\boldsymbol{(cells m}\boldsymbol{L}^{\boldsymbol{-1}}\boldsymbol{)}$ | $\boldsymbol{\eta}$ | $\boldsymbol{\sigma}$ | $\boldsymbol{-LL}$ |
| --- | --- | --- | --- | --- | --- | --- | --- | --- |
| **RGb13** | 1.00E-04 | 0.89 | 3398 | 0.35 | 1.28E+01 | 6.79E-04 | 0.35 | 10.29 |
| **RLb13** | 1.00E-03 | 0.99 | 4402 | 0.80 | 2.01E-01 | 1.60E-03 | 0.35 | 10.18 |
| **ROw8** | 1.00E-04 | 0.93 | 8000 | 0.97 | 2.82E+00 | 4.26E-03 | 0.38 | 10.45 |
| **RVy10** | 1.00E-03 | 0.94 | 8000 | 2.42 | 1.14E-02 | 1.44E-02 | 0.38 | 15.64 |
| **RKq11** | 1.00E-03 | 0.93 | 8000 | 1.67 | 3.74E-03 | 1.08E-02 | 0.37 | 15.09 |
| **RBv13** | 1.00E-03 | 0.97 | 7233 | 0.60 | 7.84E-03 | 1.11E-07 | 0.44 | 21.35 |
| **RWj14** | 1.00E-02 | 0.95 | 2208 | 1.01 | 1.35E-01 | 2.59E-04 | 0.40 | 16.50 |
| **RYF14** | 1.00E-02 | 0.97 | 3413 | 3.24 | 9.29E-01 | 1.71E-03 | 0.27 | 6.50 |
| **RAz12** | 1.00E-02 | 0.89 | 4004 | 50.00 | 8.11E+00 | 5.53E-03 | 0.46 | 28.63 |
| **RSj14** | 1.00E-02 | 0.91 | 4488 | 1.05 | 1.78E-02 | 2.22E-03 | 0.35 | 18.30 |
| **RDh10** | 1.00E-02 | 0.95 | 2695 | 3.72 | 1.99E+00 | 1.08E-03 | 0.42 | 25.55 |
| **RLc10** | 1.00E-02 | 0.95 | 8000 | 5.90 | 5.00E+01 | 6.67E-03 | 0.37 | 19.58 |
| **ROn13** | 1.00E-01 | 0.93 | 8000 | 36.47 | 5.00E+01 | 9.71E-03 | 0.39 | 20.99 |
