# Supplementary material for: CD8+ lymphocyte control of SIV infection during antiretroviral therapy
Source: PLoS Pathog. 2018 Oct 11;14(10):e1007350. doi: 10.1371/journal.ppat.1007350 (PMC6199003; doi:10.1371/journal.ppat.1007350)
Supplement: S7 Table — (DOCX) [file ppat.1007350.s009.docx]

**SI Table 7. Estimated parameter values for the CTL-VC model without cytolytic effects.**

| RM | $\boldsymbol{\alpha}_{\boldsymbol{L}}$ | $\boldsymbol{p}$ ($\boldsymbol{virions cel}\boldsymbol{l}^{\boldsymbol{-1}}\boldsymbol{d}^{\boldsymbol{-1}}$) | $\boldsymbol{d}_{\boldsymbol{E}}$ ($\boldsymbol{cells m}\boldsymbol{L}^{\boldsymbol{-1}}\boldsymbol{d}^{\boldsymbol{-1}}$) | $\boldsymbol{K}_{\boldsymbol{B}}\boldsymbol{(cells m}\boldsymbol{L}^{\boldsymbol{-1}}\boldsymbol{)}$ | $\boldsymbol{\eta}$ | $\boldsymbol{\sigma}$ | $\boldsymbol{-LL}$ |
| --- | --- | --- | --- | --- | --- | --- | --- |
| RGb13 | 6.02E-05 | 3983 | 0.34 | 3.60E-01 | 8.19E-04 | 0.48 | 15.77 |
| RLb13 | 1.61E-04 | 4943 | 0.51 | 2.57E-01 | 1.51E-03 | 0.38 | 11.55 |
| ROw8 | 3.05E-04 | 4656 | 0.46 | 2.46E-01 | 1.36E-03 | 0.37 | 10.66 |
| RVy10 | 3.26E-04 | 3942 | 1.55 | 5.00E+01 | 1.53E-03 | 0.40 | 16.89 |
| RKq11 | 6.74E-04 | 8000 | 2.81 | 1.55E-03 | 2.11E-02 | 0.41 | 17.90 |
| RBv13 | 4.37E-03 | 1940 | 0.23 | 1.00E-03 | 3.37E-06 | 0.46 | 22.94 |
| RWj14 | 6.93E-03 | 2313 | 0.45 | 6.38E-02 | 2.24E-04 | 0.37 | 15.16 |
| RYF14 | 6.34E-03 | 2211 | 0.80 | 8.07E-01 | 3.65E-05 | 0.28 | 7.58 |
| RAz12 | 1.06E-02 | 4255 | 26.01 | 4.80E+00 | 5.72E-03 | 0.46 | 28.37 |
| RSj14 | 3.43E-03 | 5742 | 4.17 | 1.76E-02 | 1.18E-02 | 0.30 | 12.96 |
| RDh10 | 5.34E-03 | 2394 | 3.49 | 1.94E+00 | 7.84E-04 | 0.42 | 25.47 |
| RLc10 | 1.74E-02 | 3682 | 4.17 | 2.47E+00 | 2.39E-03 | 0.32 | 14.34 |
| ROn13 | 8.56E-02 | 4954 | 33.06 | 5.00E+01 | 5.02E-03 | 0.42 | 24.49 |
