# Supplementary material for: CD8+ lymphocyte control of SIV infection during antiretroviral therapy
Source: PLoS Pathog. 2018 Oct 11;14(10):e1007350. doi: 10.1371/journal.ppat.1007350 (PMC6199003; doi:10.1371/journal.ppat.1007350)
Supplement: S8 Table — (DOCX) [file ppat.1007350.s010.docx]

**SI Table 8. Estimated parameter values for the CTL-VC model without non-cytolytic effects.**

| RM | $\boldsymbol{\alpha}_{\boldsymbol{L}}$ | $\boldsymbol{p}$ ($\boldsymbol{virions cel}\boldsymbol{l}^{\boldsymbol{-1}}\boldsymbol{d}^{\boldsymbol{-1}}$) | $\boldsymbol{d}_{\boldsymbol{E}}$ ($\boldsymbol{cells m}\boldsymbol{L}^{\boldsymbol{-1}}\boldsymbol{d}^{\boldsymbol{-1}}$) | $\boldsymbol{K}_{\boldsymbol{B}}\boldsymbol{(cells m}\boldsymbol{L}^{\boldsymbol{-1}}\boldsymbol{)}$ | $\boldsymbol{\sigma}$ | $\boldsymbol{-LL}$ |
| --- | --- | --- | --- | --- | --- | --- |
| RGb13 | 3.05E-04 | 1815 | 0.45 | 9.11E-02 | 0.40 | 12.09 |
| RLb13 | 7.84E-04 | 1922 | 0.57 | 5.93E-02 | 0.44 | 14.53 |
| ROw8 | 1.55E-05 | 5225 | 0.58 | 4.62E+01 | 0.53 | 17.70 |
| RVy10 | 1.64E-03 | 1906 | 1.13 | 5.58E-02 | 0.45 | 18.98 |
| RKq11 | 4.39E-03 | 1923 | 0.69 | 6.56E-02 | 0.47 | 21.31 |
| RBv13 | 1.35E-03 | 3177 | 1.05 | 3.43E-02 | 0.48 | 23.86 |
| RWj14 | 7.81E-03 | 1880 | 0.77 | 8.26E-02 | 0.43 | 18.79 |
| RYF14 | 5.76E-03 | 2271 | 1.28 | 2.61E-01 | 0.33 | 12.87 |
| RAz12 | 1.30E-02 | 2071 | 1.60 | 3.18E-01 | 0.57 | 37.31 |
| RSj14 | 3.37E-03 | 3185 | 1.17 | 4.61E-02 | 0.40 | 22.01 |
| RDh10 | 6.93E-03 | 1951 | 1.67 | 6.60E-01 | 0.47 | 29.00 |
| RLc10 | 7.69E-03 | 3607 | 1.27 | 3.15E-01 | 0.46 | 27.78 |
| ROn13 | 5.74E-02 | 4249 | 1.28 | 2.82E+00 | 0.55 | 35.95 |
