# Supplementary material for: CD8+ lymphocyte control of SIV infection during antiretroviral therapy
Source: PLoS Pathog. 2018 Oct 11;14(10):e1007350. doi: 10.1371/journal.ppat.1007350 (PMC6199003; doi:10.1371/journal.ppat.1007350)
Supplement: S9 Table — (DOCX) [file ppat.1007350.s011.docx]

**SI Table 9. Estimated parameter values for the CTL-VC long-lived infected cell model with** $\boldsymbol{\beta}_{\boldsymbol{M}}\boldsymbol{=6\times}\boldsymbol{10}^{\boldsymbol{-9}}\boldsymbol{mL}\boldsymbol{d}^{\boldsymbol{-1}}$**,** $\boldsymbol{\delta}_{\boldsymbol{M}}\boldsymbol{=0.20}\boldsymbol{d}^{\boldsymbol{-1}}$ **and** $\boldsymbol{m}_{\boldsymbol{M}}\boldsymbol{=3\times}\boldsymbol{10}^{\boldsymbol{-5}}\boldsymbol{mL cel}\boldsymbol{l}^{\boldsymbol{-1}}\boldsymbol{d}^{\boldsymbol{-1}}$**.**

| RM | $\boldsymbol{\alpha}_{\boldsymbol{L}}$ | $\boldsymbol{p}$ ($\boldsymbol{virions cel}\boldsymbol{l}^{\boldsymbol{-1}}\boldsymbol{d}^{\boldsymbol{-1}}$) | $\boldsymbol{d}_{\boldsymbol{E}}$ ($\boldsymbol{cells m}\boldsymbol{L}^{\boldsymbol{-1}}\boldsymbol{d}^{\boldsymbol{-1}}$) | $\boldsymbol{K}_{\boldsymbol{B}}\boldsymbol{(cells m}\boldsymbol{L}^{\boldsymbol{-1}}\boldsymbol{)}$ | $\boldsymbol{\eta}$ | $\boldsymbol{\sigma}$ | $\boldsymbol{-LL}$ |
| --- | --- | --- | --- | --- | --- | --- | --- |
| RGb13 | 8.56E-05 | 3893 | 0.35 | 1.79E+01 | 8.92E-04 | 0.34 | 9.89 |
| RLb13 | 3.50E-04 | 4123 | 0.63 | 2.11E-01 | 1.17E-03 | 0.34 | 9.53 |
| ROw8 | 3.24E-04 | 4709 | 0.63 | 3.54E-01 | 1.54E-03 | 0.34 | 8.98 |
| RVy10 | 5.76E-04 | 3511 | 0.95 | 1.07E+00 | 1.07E-03 | 0.40 | 16.28 |
| RKq11 | 1.14E-03 | 3947 | 1.03 | 3.65E-03 | 2.29E-03 | 0.36 | 15.33 |
| RBv13 | 4.31E-03 | 2043 | 0.96 | 7.04E-02 | 4.66E-05 | 0.47 | 23.02 |
| RWj14 | 9.70E-03 | 1944 | 0.68 | 7.66E-02 | 5.56E-05 | 0.39 | 15.50 |
| RYF14 | 6.61E-03 | 2403 | 2.28 | 5.05E-01 | 3.64E-04 | 0.28 | 7.45 |
| RAz12 | 1.26E-02 | 3521 | 17.17 | 4.85E+00 | 3.30E-03 | 0.47 | 29.29 |
| RSj14 | 5.11E-03 | 3958 | 1.27 | 1.03E-02 | 2.06E-03 | 0.30 | 11.99 |
| RDh10 | 6.26E-03 | 2305 | 4.12 | 2.60E+00 | 6.54E-04 | 0.41 | 24.84 |
| RLc10 | 2.45E-02 | 3475 | 3.63 | 2.25E+00 | 1.88E-03 | 0.34 | 16.71 |
| ROn13 | 9.89E-02 | 4912 | 26.23 | 5.00E+01 | 4.39E-03 | 0.39 | 20.69 |
