# Supplementary material for: CD8+ lymphocyte control of SIV infection during antiretroviral therapy
Source: PLoS Pathog. 2018 Oct 11;14(10):e1007350. doi: 10.1371/journal.ppat.1007350 (PMC6199003; doi:10.1371/journal.ppat.1007350)
Supplement: S10 Table — (DOCX) [file ppat.1007350.s012.docx]

**SI Table 10. Estimated parameter values for the eclipse-CTL-VC model.**

| RM | $\boldsymbol{\alpha}_{\boldsymbol{L}}$ | $\boldsymbol{p}$ ($\boldsymbol{virions cel}\boldsymbol{l}^{\boldsymbol{-1}}\boldsymbol{d}^{\boldsymbol{-1}}$) | $\boldsymbol{d}_{\boldsymbol{E}}$ ($\boldsymbol{cells m}\boldsymbol{L}^{\boldsymbol{-1}}\boldsymbol{d}^{\boldsymbol{-1}}$) | $\boldsymbol{K}_{\boldsymbol{B}}\boldsymbol{(cells m}\boldsymbol{L}^{\boldsymbol{-1}}\boldsymbol{)}$ | $\boldsymbol{\eta}$ | $\boldsymbol{t}_{\boldsymbol{E}}$ (days) | $\boldsymbol{\sigma}$ | $\boldsymbol{-LL}$ |
| --- | --- | --- | --- | --- | --- | --- | --- | --- |
| RGb13 | 3.65E-05 | 23,728 | 0.25 | 5.15E+00 | 8.59E-04 | 1.44 | 0.28 | 6.64 |
| RLb13 | 3.30E-04 | 16,876 | 0.25 | 5.05E-03 | 1.22E-06 | 1.82 | 0.37 | 11.71 |
| ROw8 | 7.13E-04 | 20,101 | 0.25 | 1.04E-02 | 1.85E-05 | 2.12 | 0.42 | 12.51 |
| RVy10 | 5.21E-04 | 18,178 | 0.26 | 1.24E-02 | 1.00E-07 | 2.29 | 0.44 | 18.18 |
| RKq11 | 1.26E-03 | 18,234 | 1.51 | 1.90E-02 | 6.64E-04 | 1.62 | 0.44 | 19.53 |
| RBv13 | 1.30E-03 | 16,587 | 0.77 | 8.75E-03 | 2.76E-05 | 1.83 | 0.40 | 18.65 |
| RWj14 | 3.27E-03 | 14,551 | 0.73 | 1.75E-02 | 8.24E-05 | 1.61 | 0.41 | 17.43 |
| RYF14 | 2.36E-03 | 17,060 | 0.88 | 2.95E-02 | 9.46E-06 | 1.94 | 0.29 | 8.82 |
| RAz12 | 8.68E-03 | 25,000 | 50.00 | 1.05E+00 | 6.23E-03 | 1.27 | 0.48 | 29.98 |
| RSj14 | 2.94E-03 | 20,532 | 2.76 | 8.69E-03 | 1.44E-03 | 1.54 | 0.35 | 17.53 |
| RDh10 | 2.40E-03 | 14,903 | 0.86 | 5.95E-02 | 1.00E-07 | 2.26 | 0.44 | 27.69 |
| RLc10 | 1.15E-02 | 19,265 | 1.63 | 1.59E-01 | 4.00E-04 | 2.02 | 0.34 | 16.66 |
| ROn13 | 3.22E-02 | 25,000 | 2.11 | 6.84E-01 | 2.57E-04 | 3.28 | 0.41 | 23.30 |
