# Supplementary figures and images for: CD8+ lymphocyte control of SIV infection during antiretroviral therapy
Source: PLoS Pathog. 2018 Oct 11;14(10):e1007350. doi: 10.1371/journal.ppat.1007350 (PMC6199003; doi:10.1371/journal.ppat.1007350)

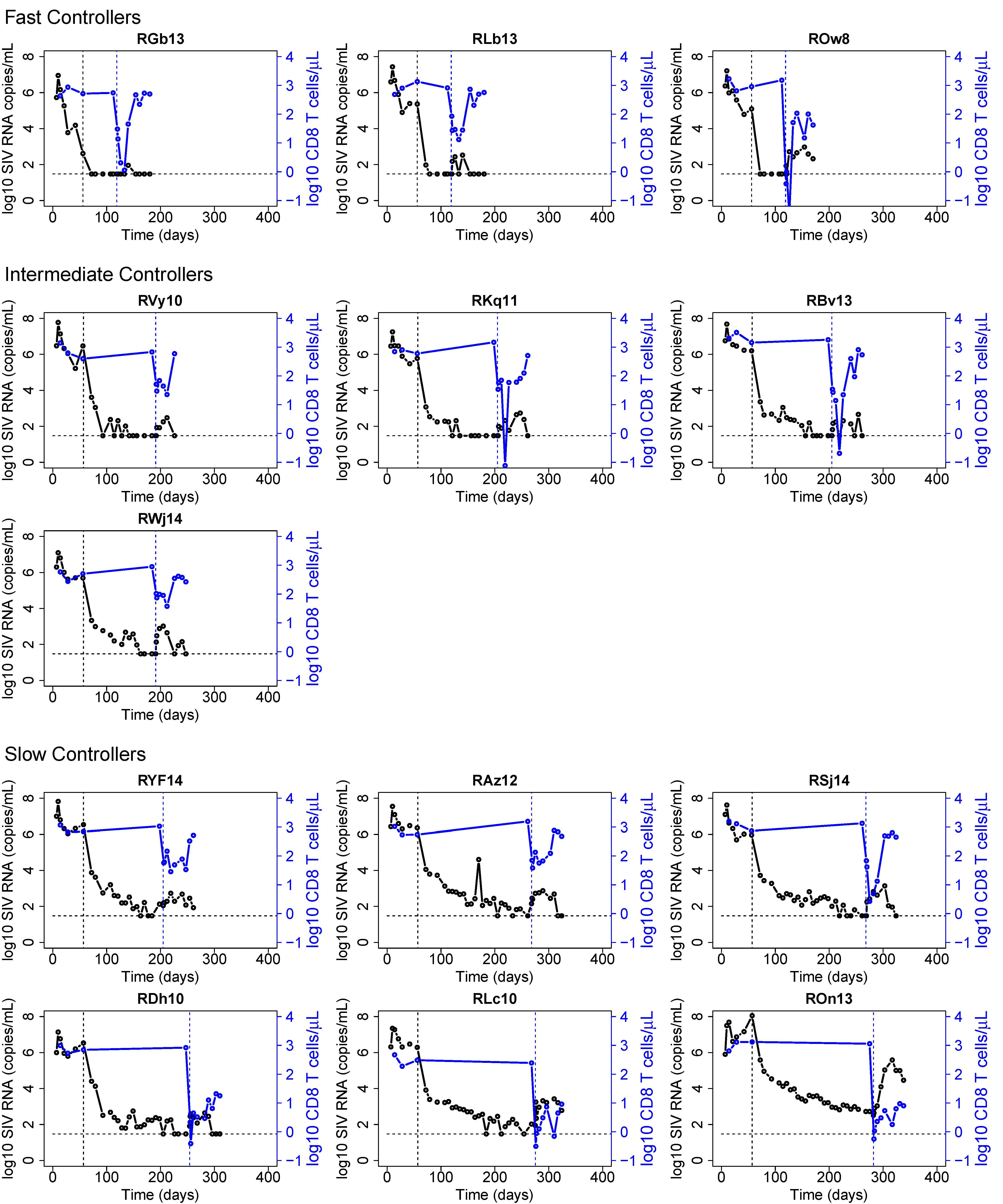

Supplement: S1 Fig — (TIF) [file ppat.1007350.s013.tif]

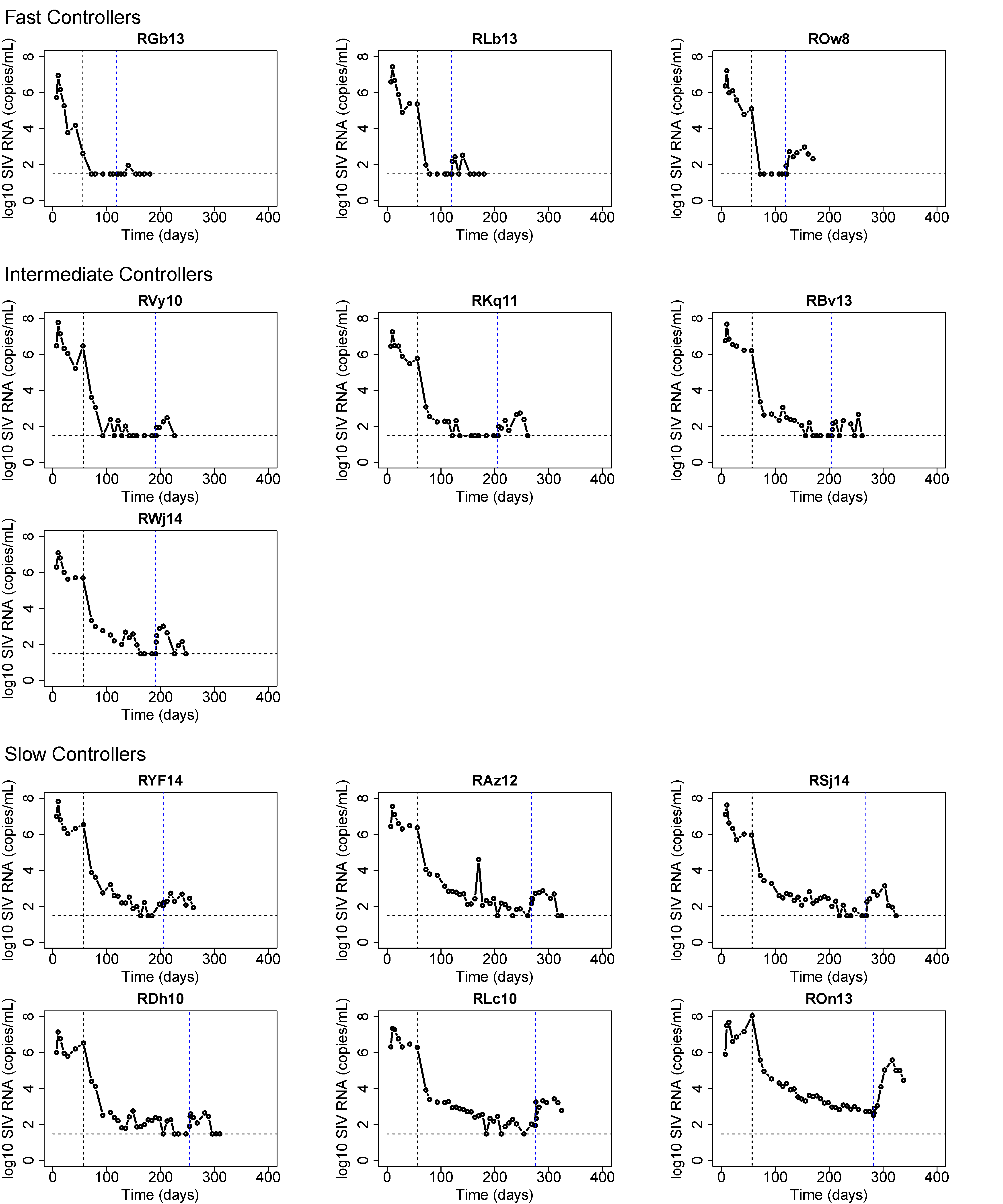

Supplement: S2 Fig — Black dots are the longitudinal VL data with connecting straight lines for eye guiding. The first vertical dashed line indicates the start time of ART, and the second vertical dashed line indicates the time of anti-CD8 antibody administration. The horizontal dashed line indicates the VL detection limit. (TIF) [file ppat.1007350.s014.tif]

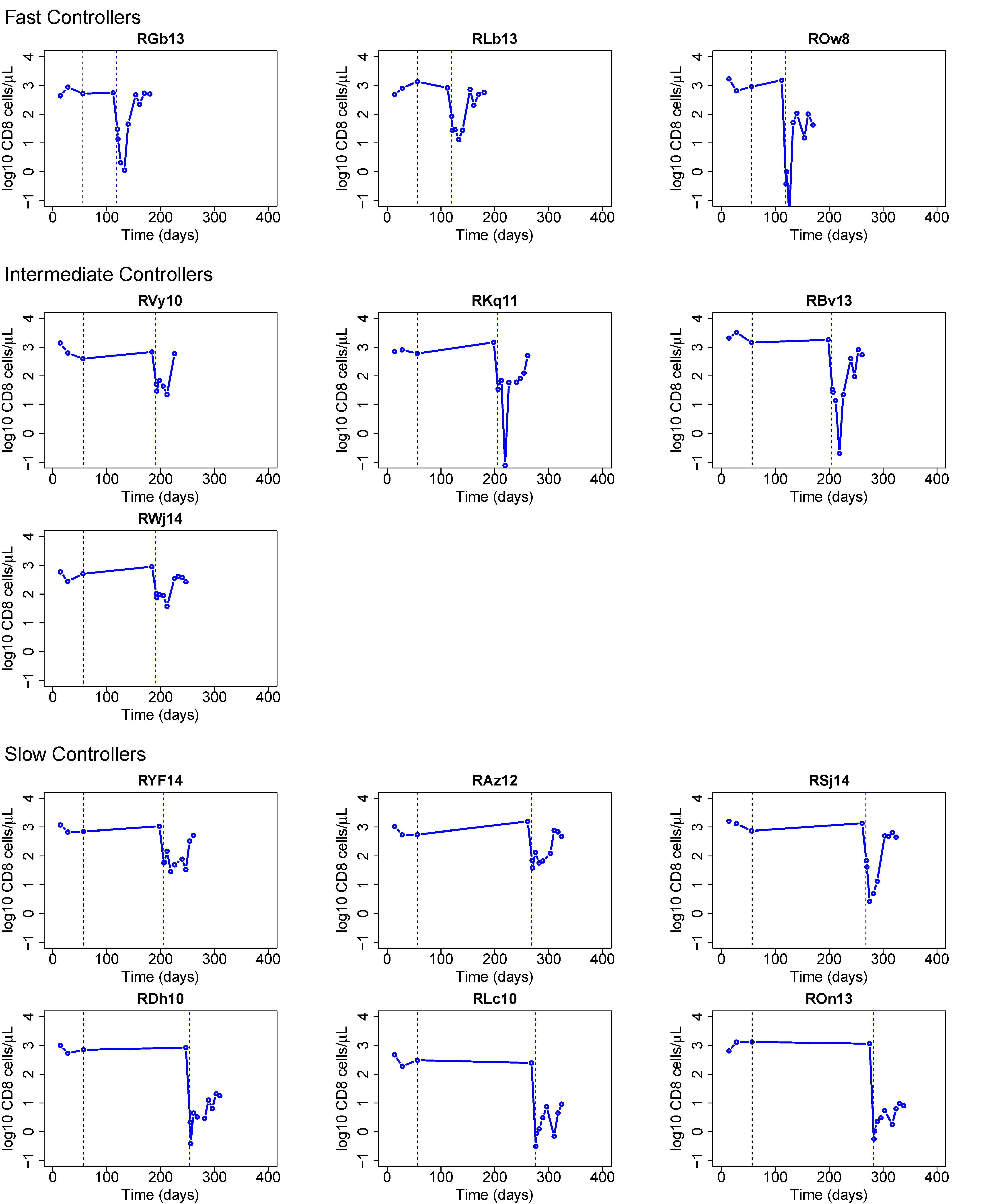

Supplement: S3 Fig — Blue dots are the longitudinal total CD8 count with connecting straight lines for eye guiding. The first vertical dashed line indicates the start time of ART, and the second vertical dashed line indicates the time of anti-CD8 antibody administration. (TIF) [file ppat.1007350.s015.tif]

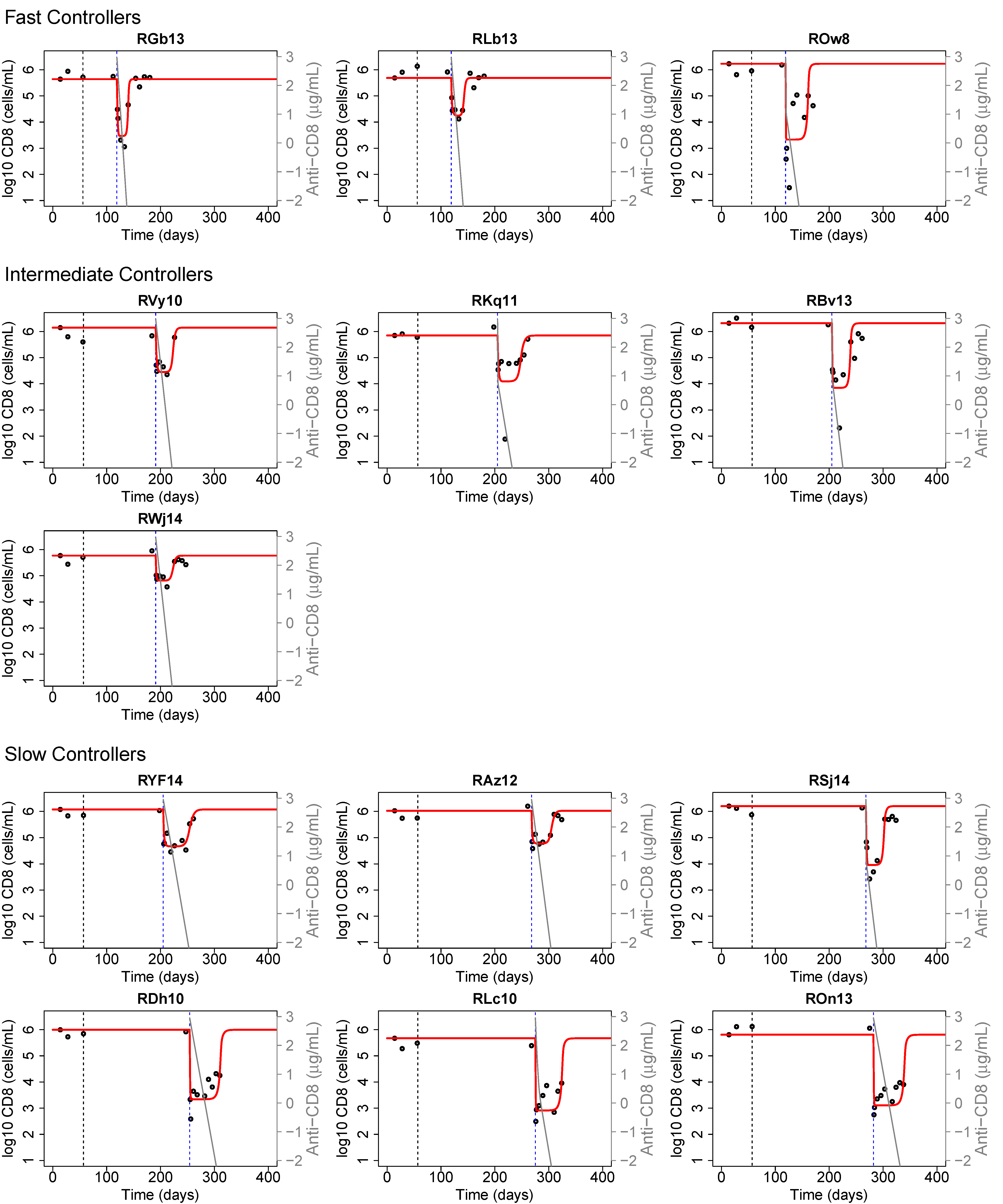

Supplement: S4 Fig — (TIF) [file ppat.1007350.s016.tif]

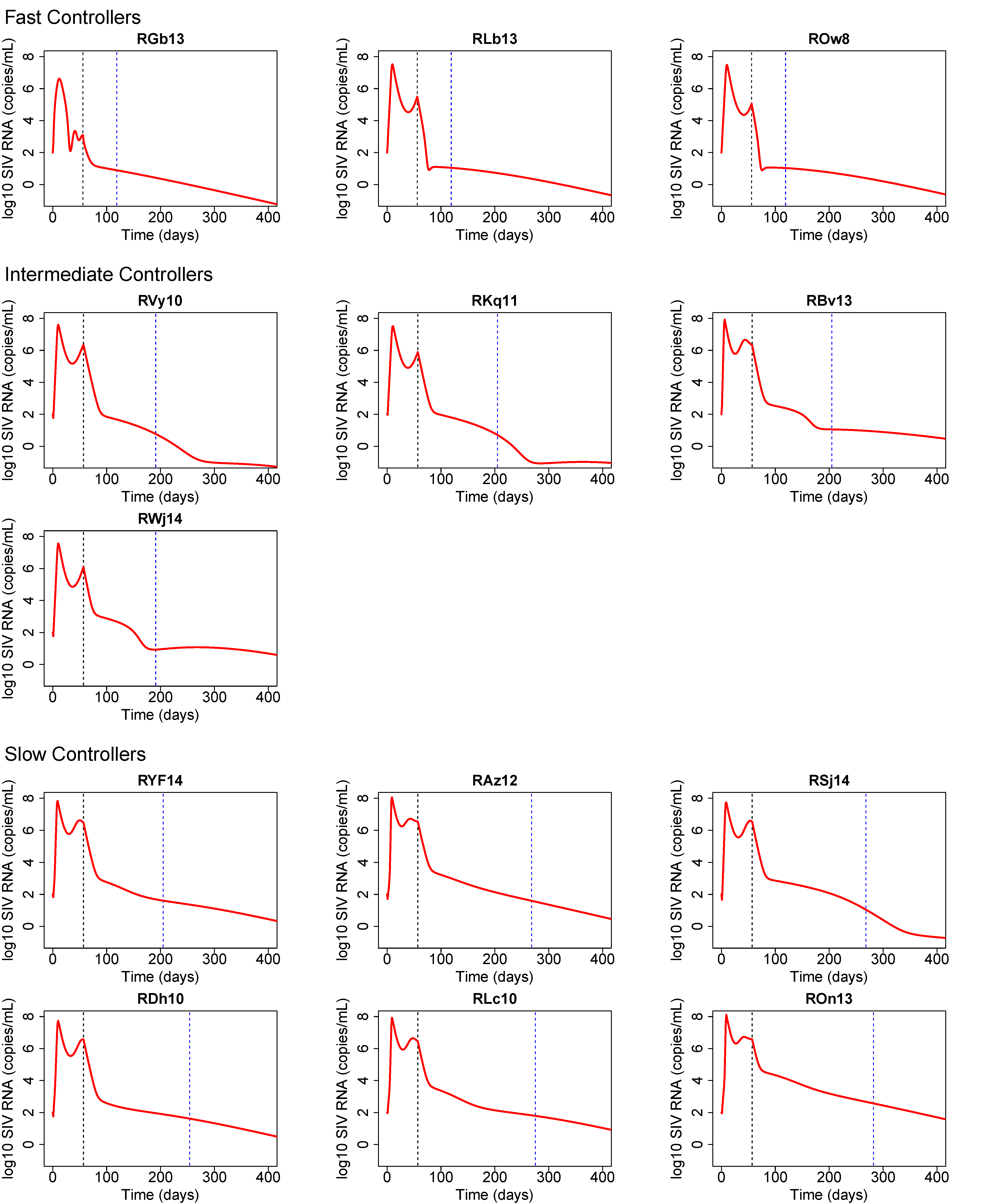

Supplement: S5 Fig — The first vertical dashed line is the start time of ART, and the second vertical dashed line is the start time of CD8 depletion in the experiment. (TIF) [file ppat.1007350.s017.tif]

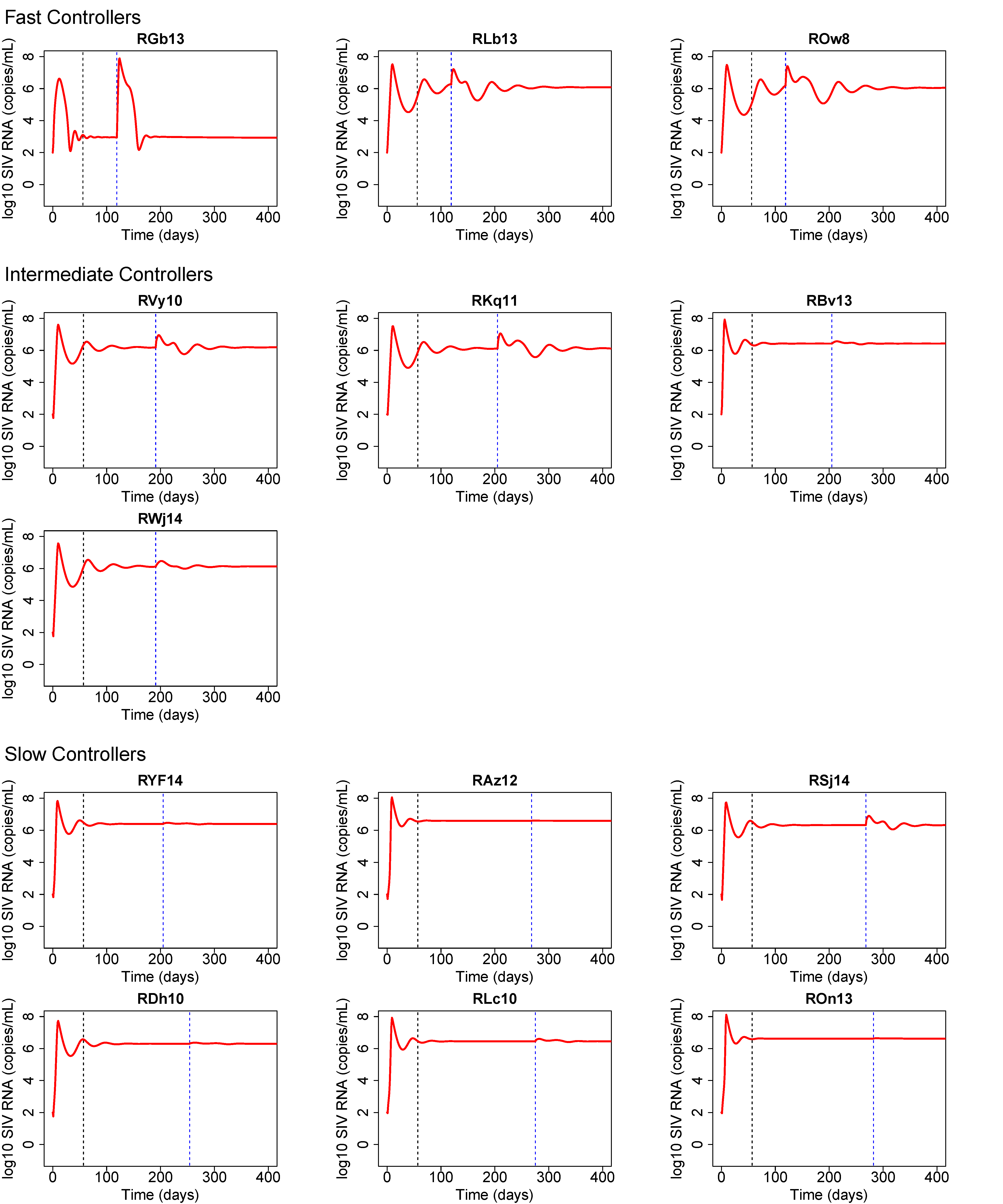

Supplement: S6 Fig — The first vertical dashed line is the start time of ART in the experiment, and the second vertical dashed line is the start time of CD8 depletion. (TIF) [file ppat.1007350.s018.tif]

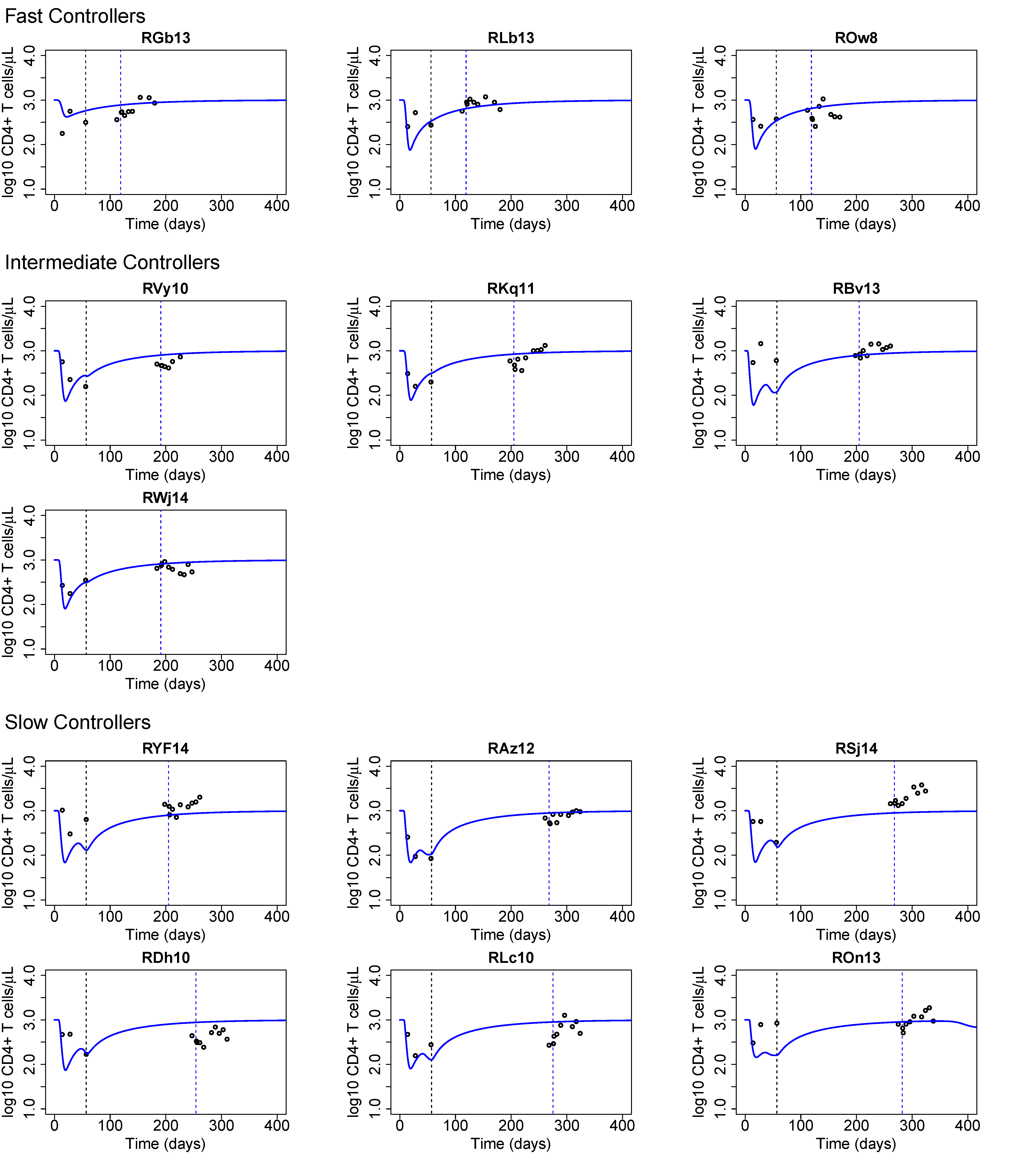

Supplement: S7 Fig — Black circles are CD4+ T cell count data from Cartwright et al. (10). Blue lines are the dynamics of total CD4+ T cell population calculated as the sum of target cell (T), latently infected cell (L) and the productively infected cell (I) from the CTL-VC model prediction. (TIF) [file ppat.1007350.s019.tif]

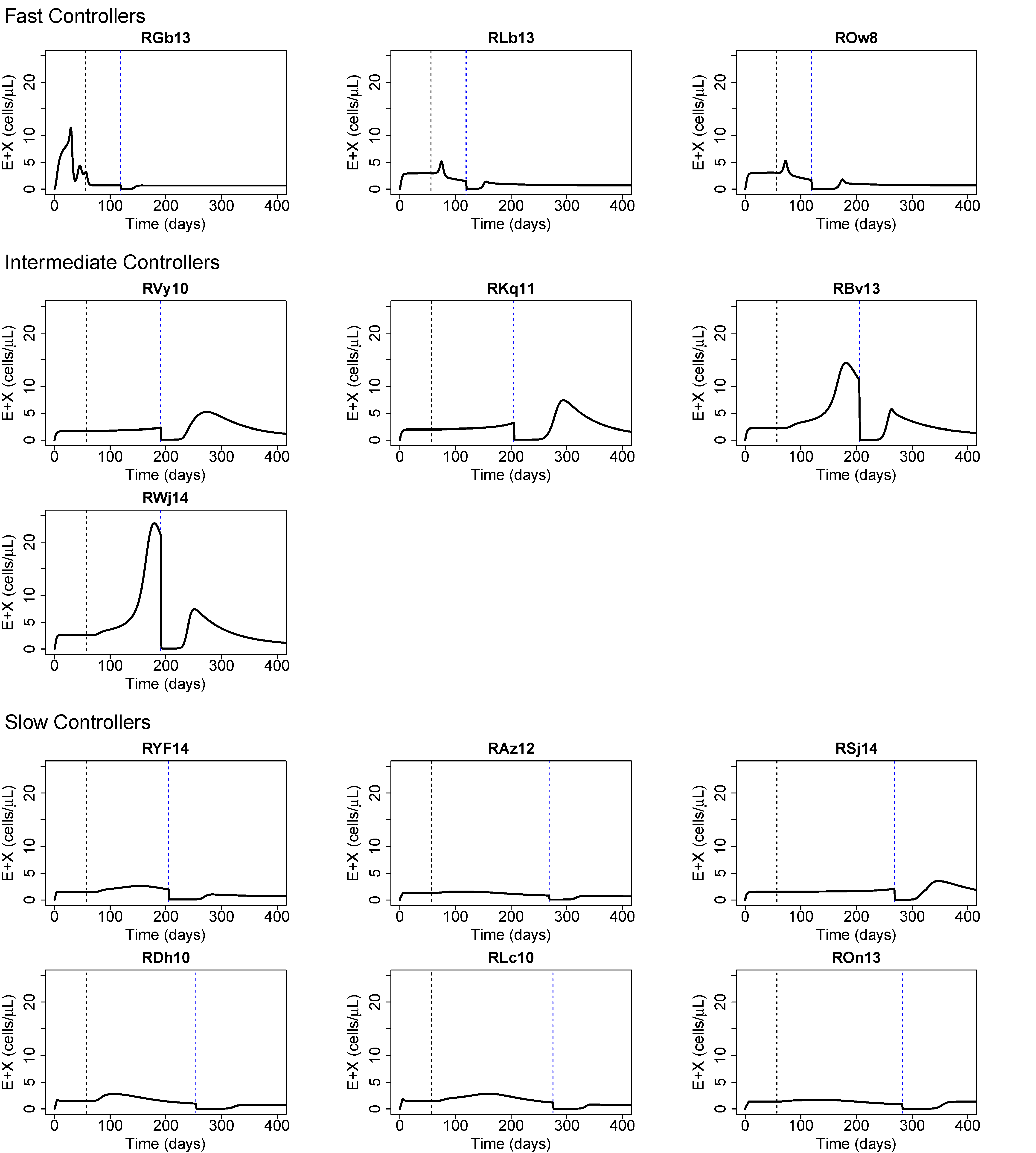

Supplement: S8 Fig — It consists of the sum of the effector cell population (E) and the exhausted cell population (X). (TIF) [file ppat.1007350.s020.tif]

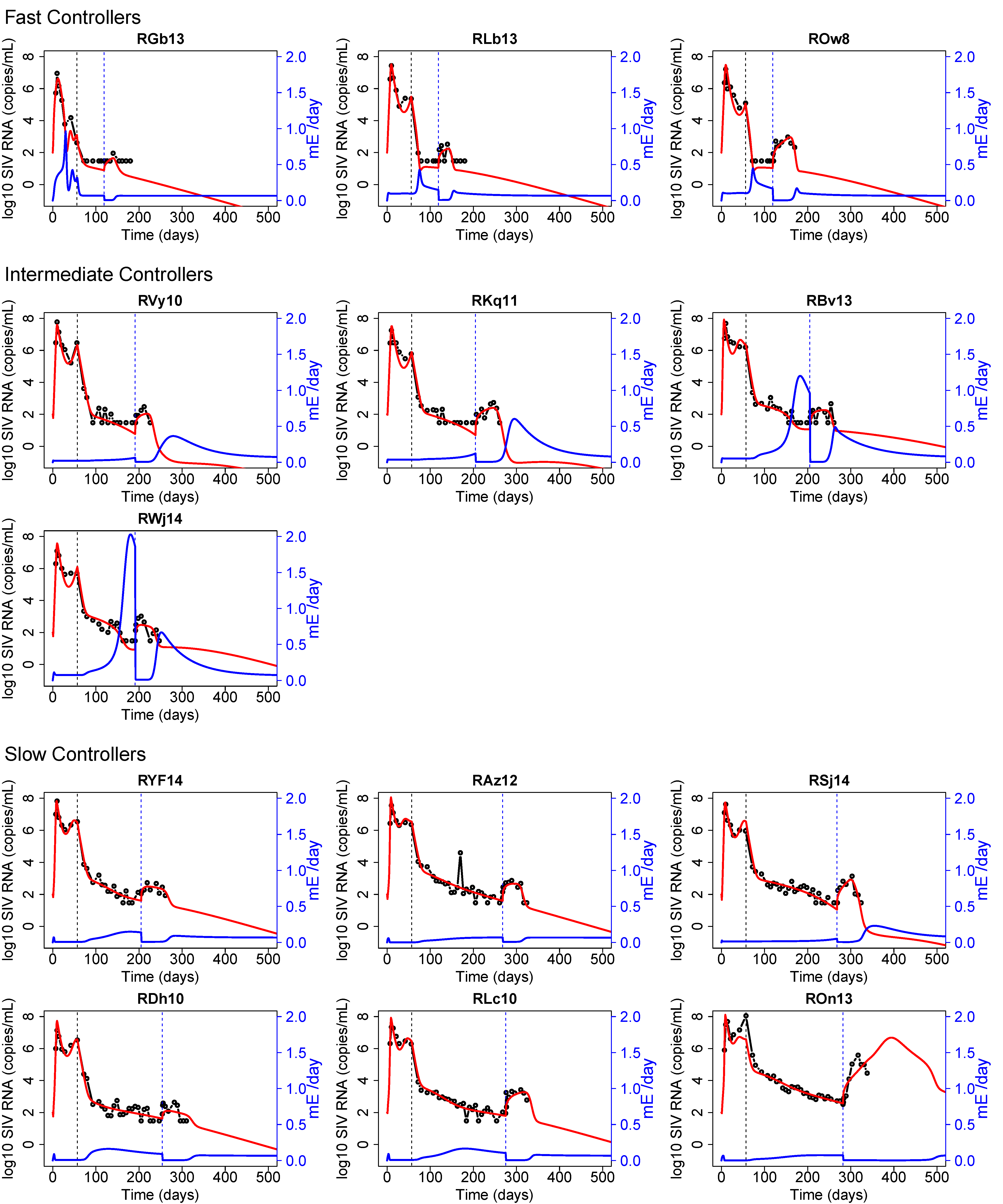

Supplement: S9 Fig — (TIF) [file ppat.1007350.s021.tif]

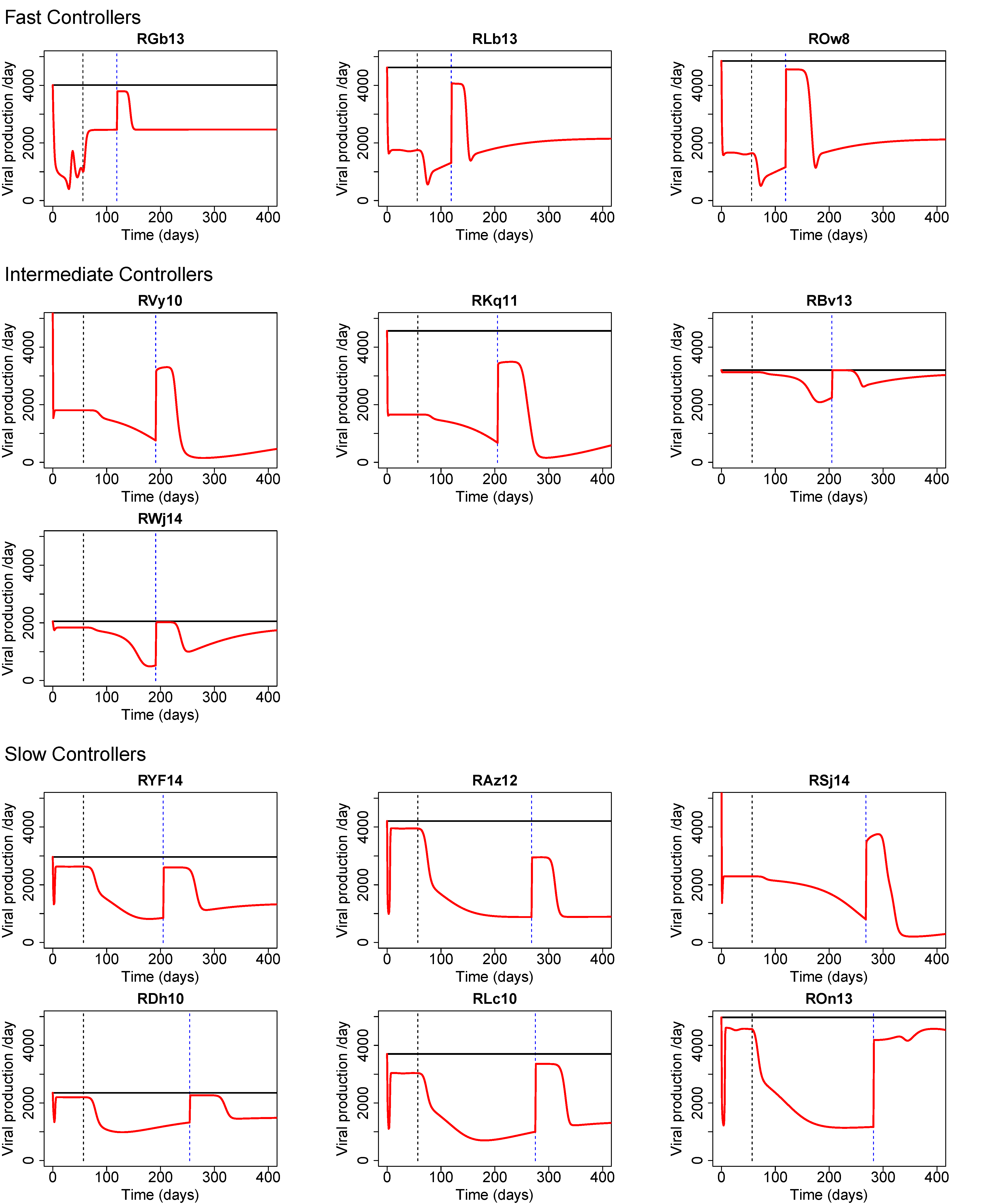

Supplement: S10 Fig — Red lines: effective viral production rate. Black lines: the maximum viral production rate p estimated from data fitting. (TIF) [file ppat.1007350.s022.tif]

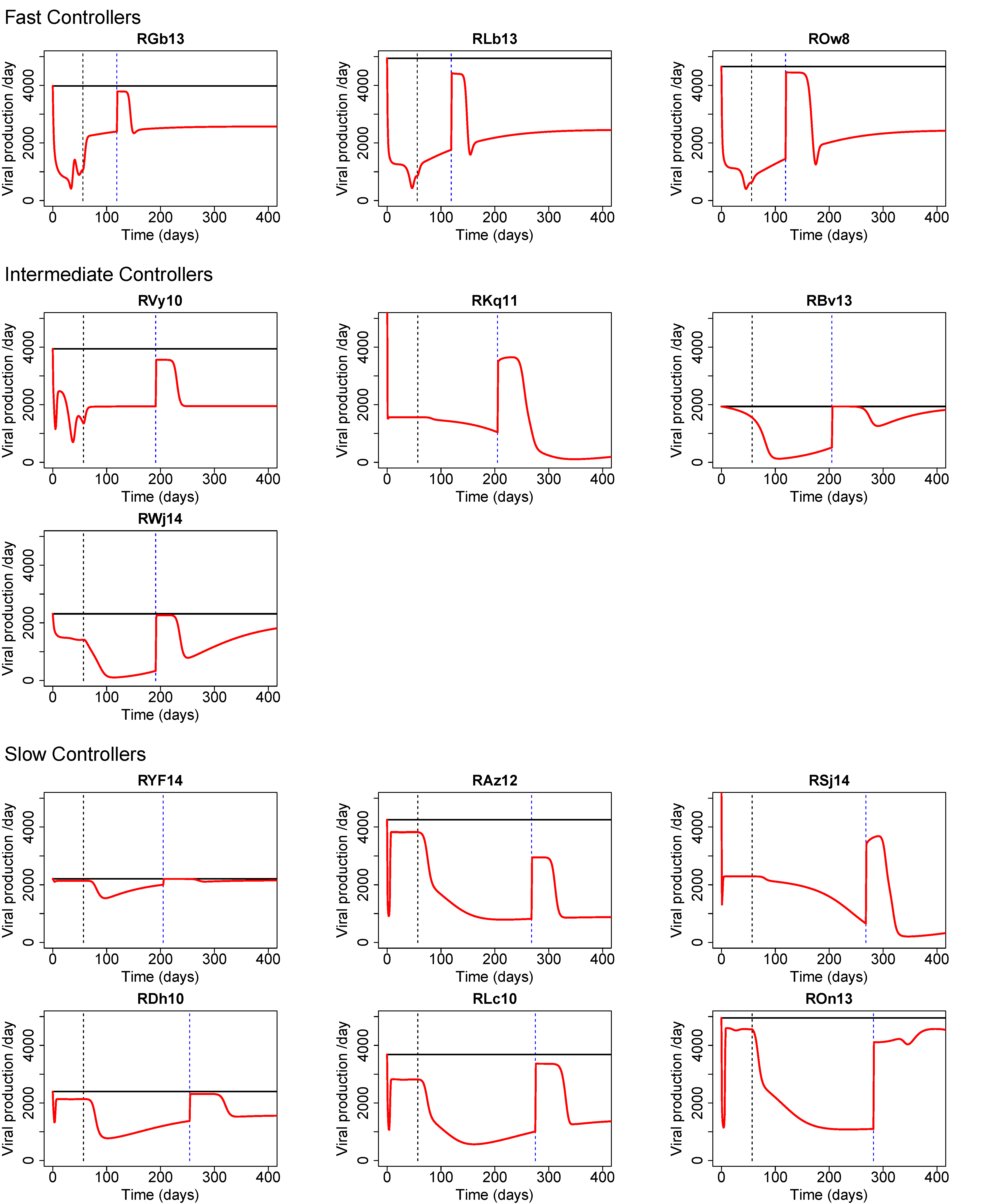

Supplement: S11 Fig — Red lines: effective viral production rate. Black lines: the maximum viral production rate p estimated from data fitting. (TIF) [file ppat.1007350.s023.tif]

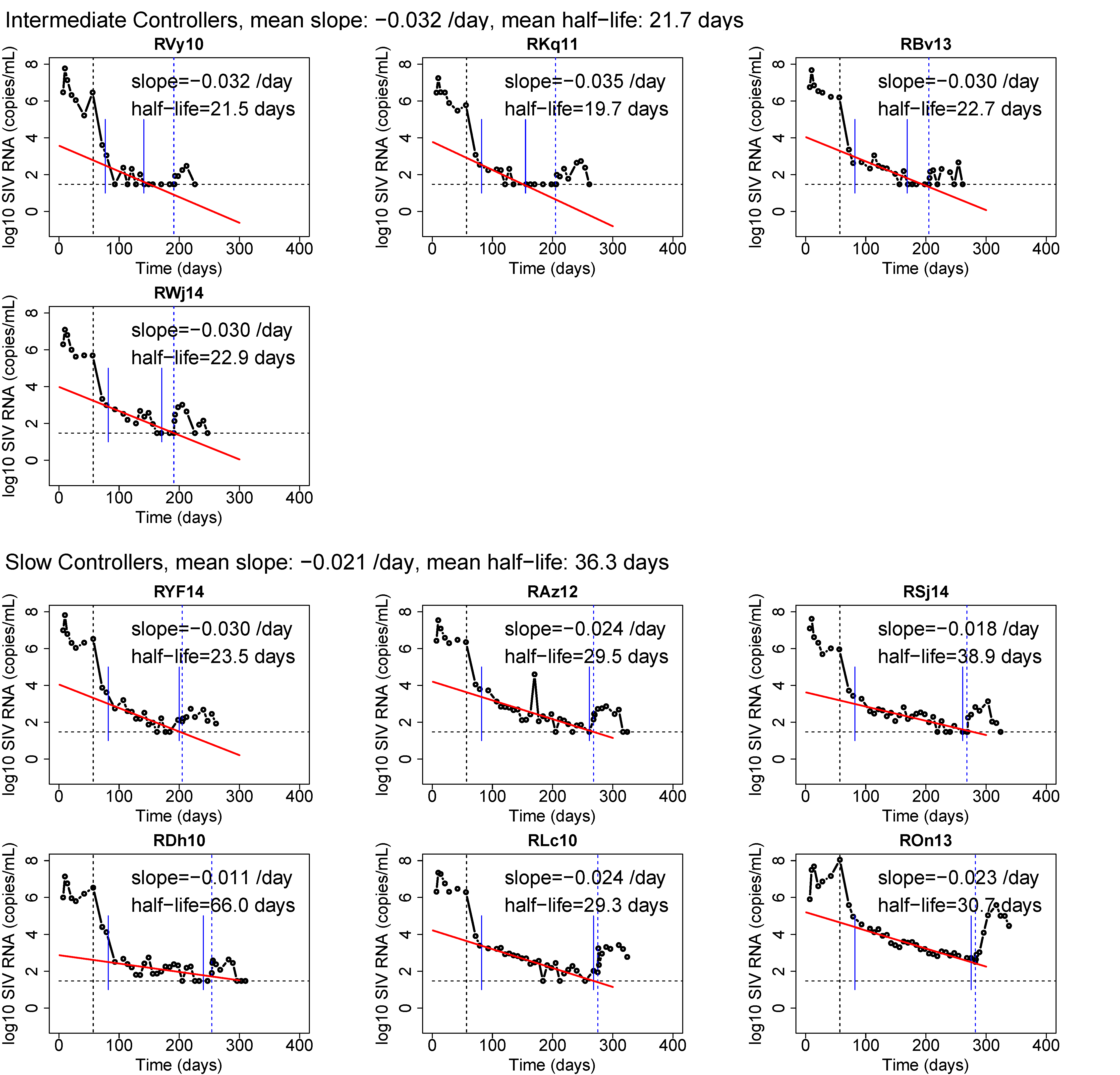

Supplement: S12 Fig — Two blue vertical lines indicate the time period for computing the second phase. Red line indicates the slope of second phase decay for each animal computed from VL data between the two blue vertical lines. (TIF) [file ppat.1007350.s024.tif]

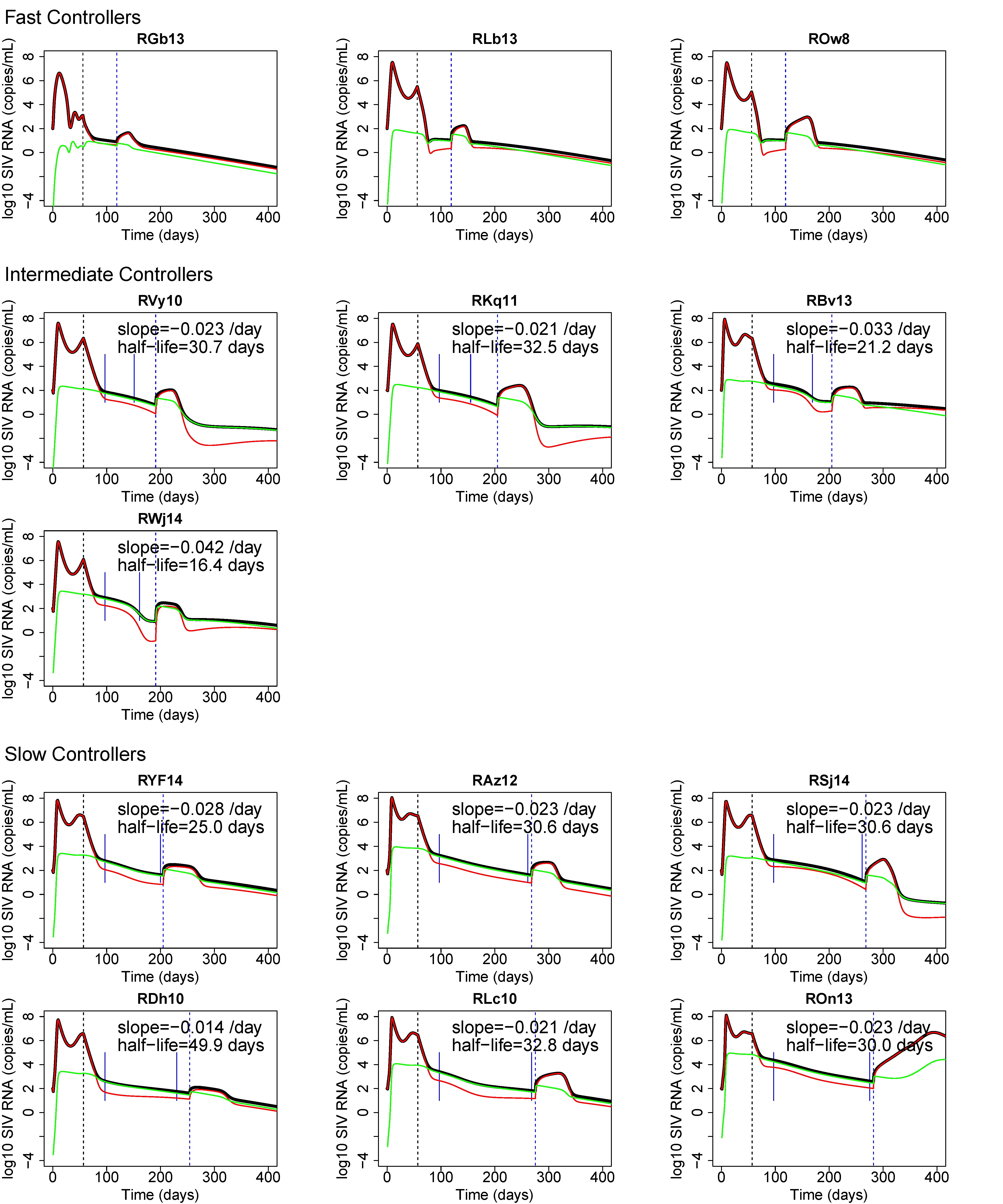

Supplement: S13 Fig — Slopes and half-lives are computed from the total viral load dynamics. (TIF) [file ppat.1007350.s025.tif]

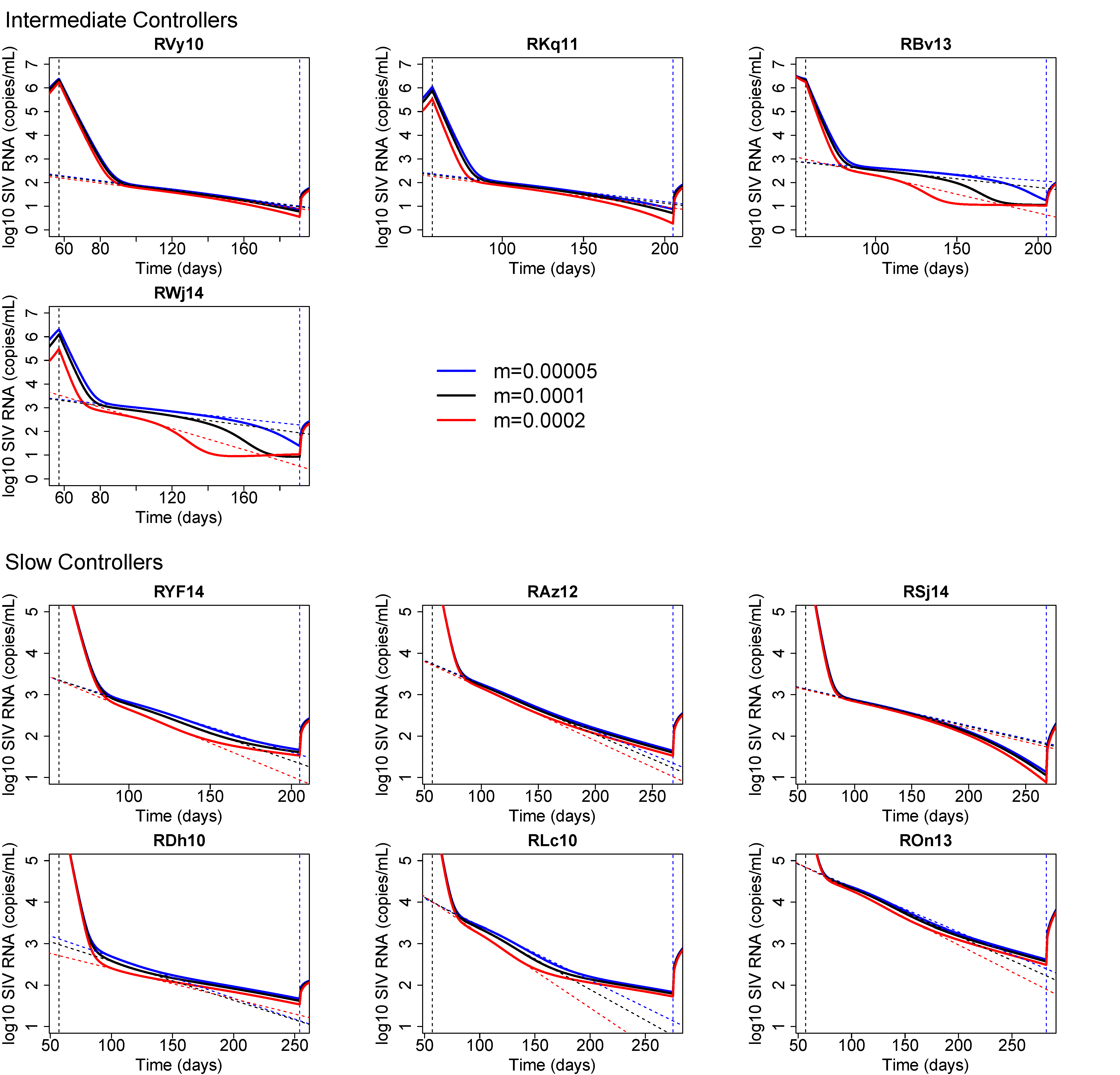

Supplement: S14 Fig — Black lines are the simulated viral load dynamics with the original value of m = 10−4 mL cell−1 d−1, while blue lines are simulations with a weaker CD8 effector cell response m = 5 × 10−5 mL cell−1 d−1 and red lines with a stronger CD8 response m = 2 × 10−4 mL cell−1 d−1. (TIF) [file ppat.1007350.s026.tif]

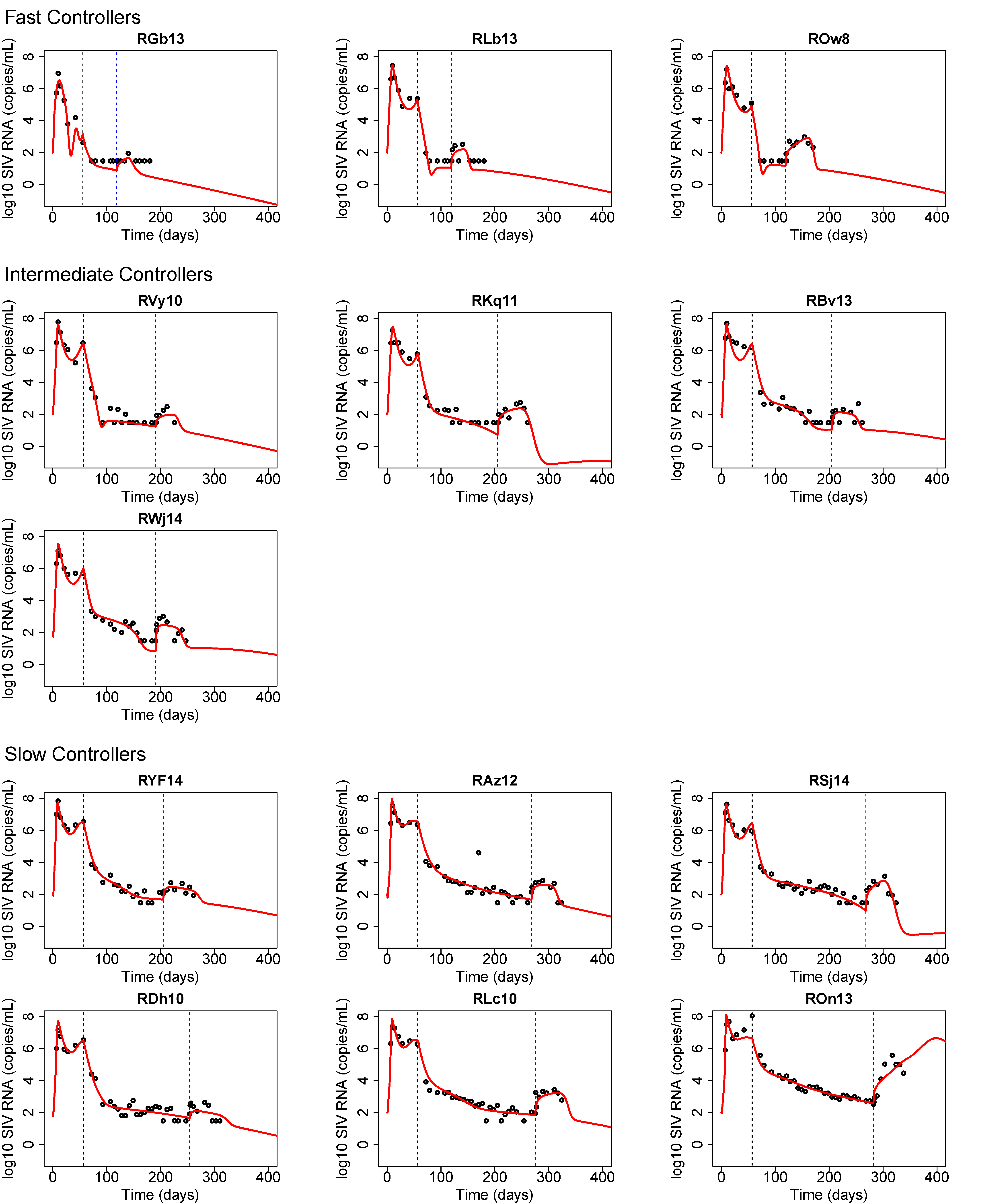

Supplement: S15 Fig — Red lines are the best model fits, and black dots are VL data points. (TIF) [file ppat.1007350.s027.tif]

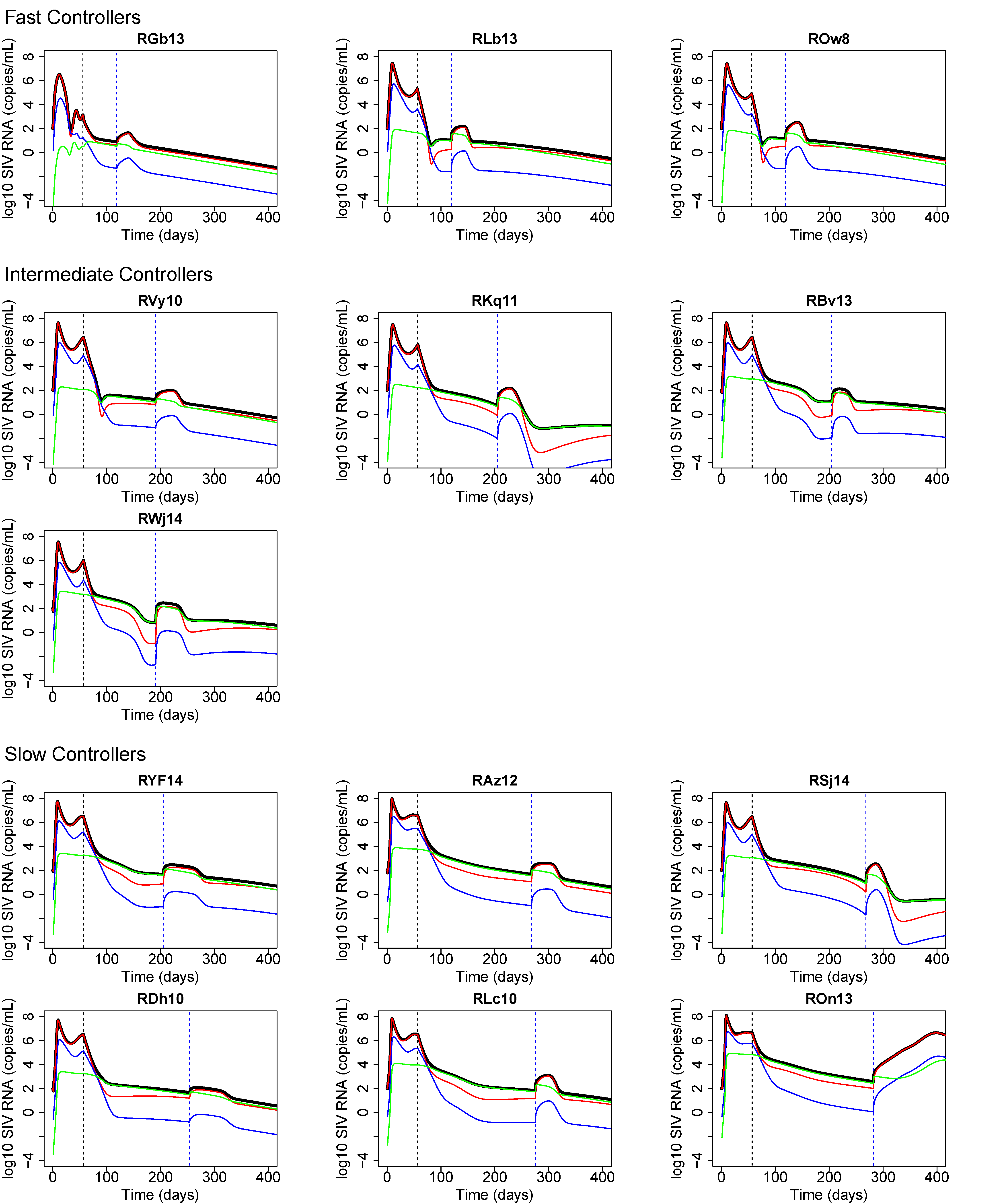

Supplement: S16 Fig — Black lines are the overall viral dynamics. (TIF) [file ppat.1007350.s028.tif]

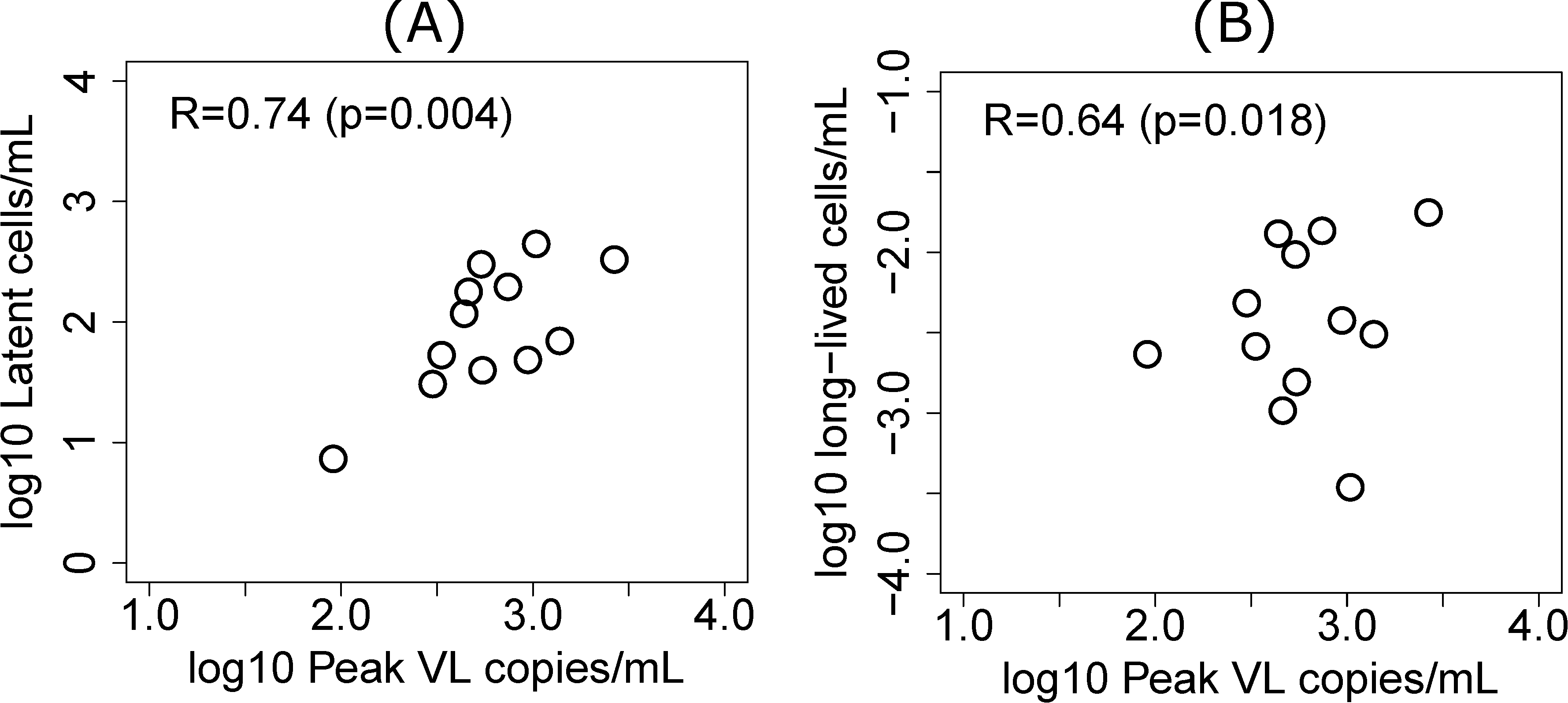

Supplement: S17 Fig — In the CTL-VC long-lived infected cell model, the correlation between (A) pre-depletion latent reservoir size and the post-depletion peak VL remains strong, while (B) the pre-depletion long-lived infected cell population also shows a correlation with the post-depletion peak VL. (TIF) [file ppat.1007350.s029.tif]

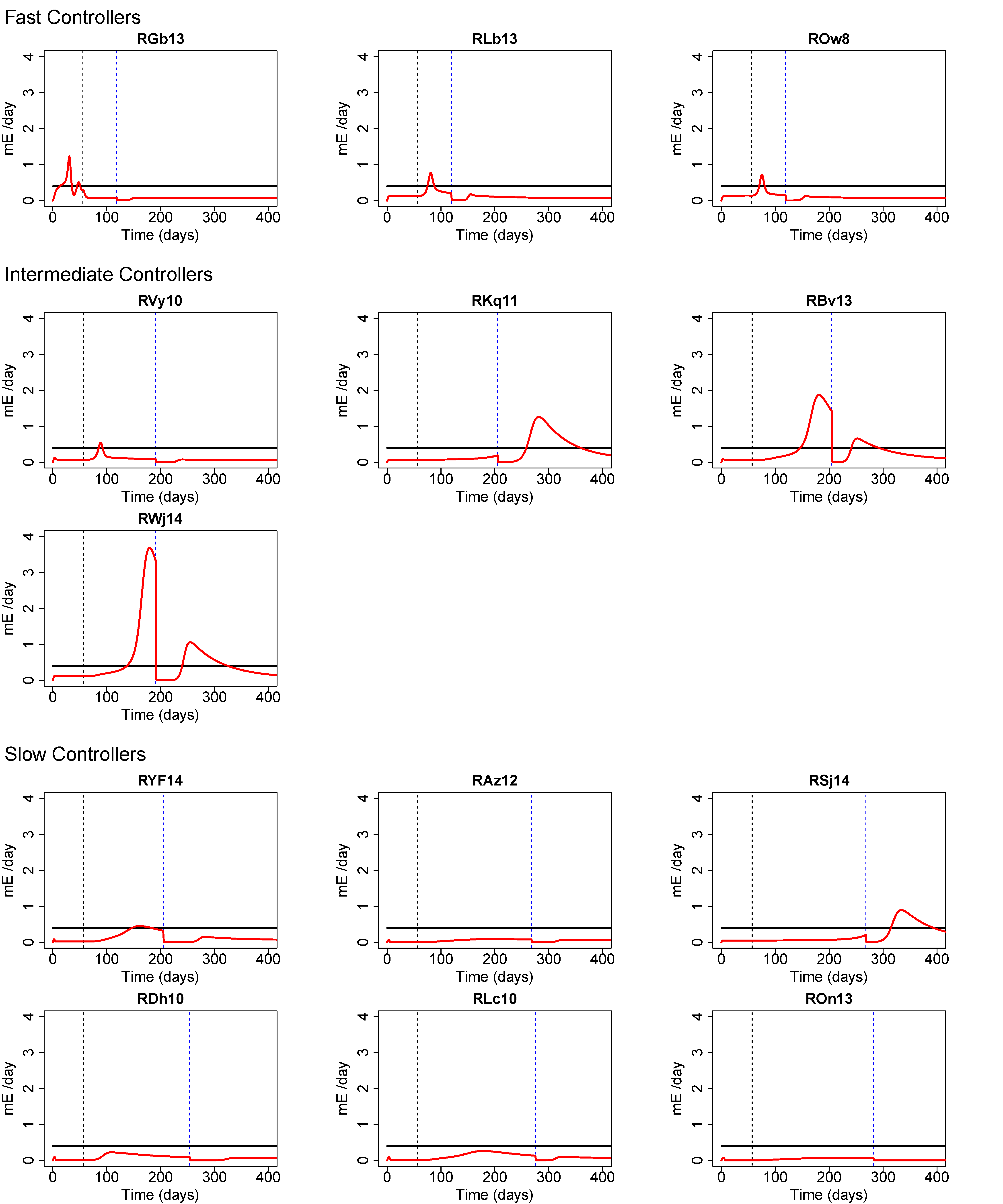

Supplement: S18 Fig — Red lines are the predicted effector cell cytolytic killing rate mE. The black horizontal lines are the fixed cytopathic death rate of infected cells, δ = 0.40 d−1. (TIF) [file ppat.1007350.s030.tif]

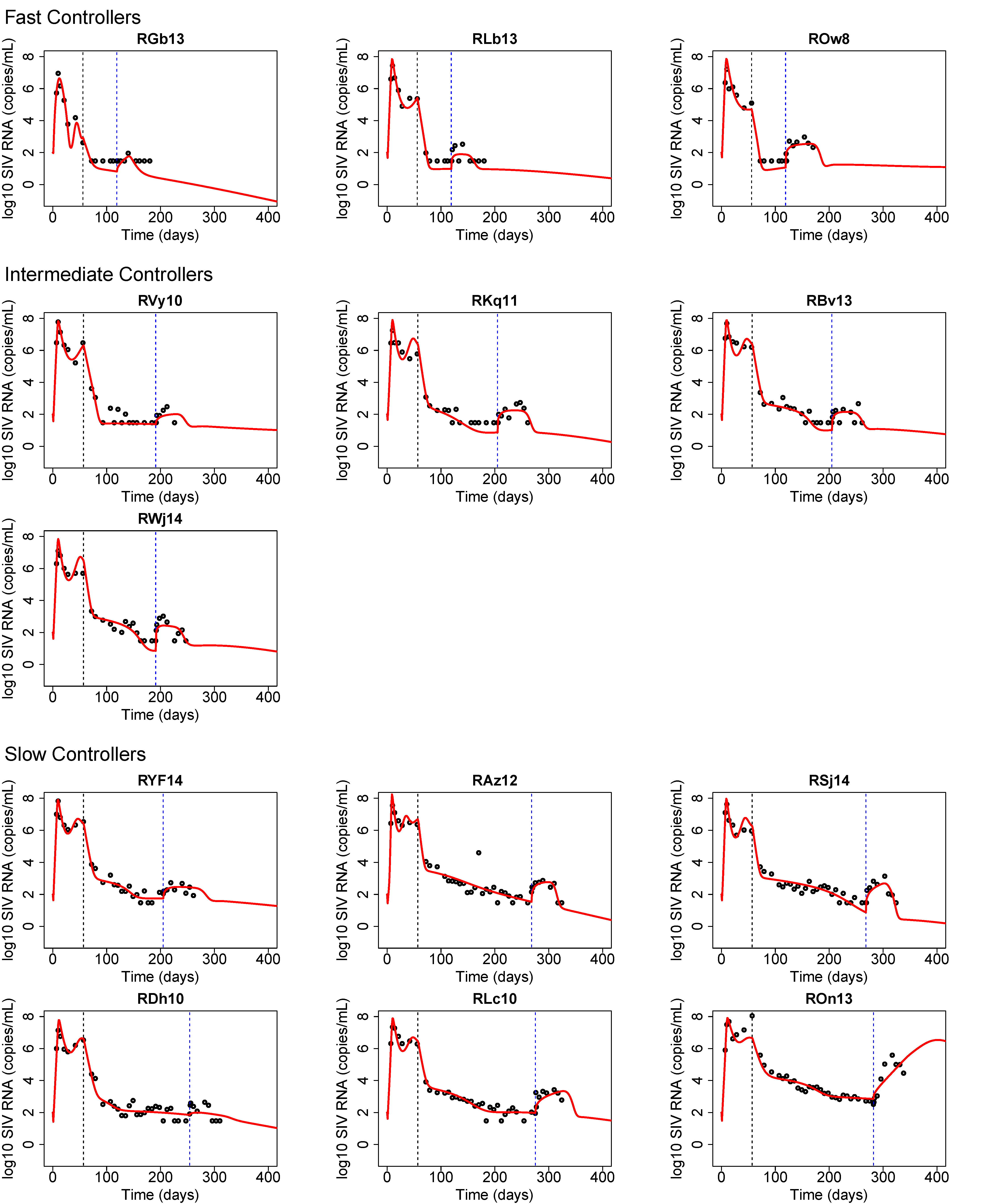

Supplement: S19 Fig — Red lines are model fits, and black dots are VL data points. (TIF) [file ppat.1007350.s031.tif]
